# The HIV-1 proviral landscape reveals that Nef contributes to HIV-1 persistence in effector memory CD4^+^ T cells

**DOI:** 10.1172/JCI154422

**Published:** 2022-04-01

**Authors:** Gabriel Duette, Bonnie Hiener, Hannah Morgan, Fernando G. Mazur, Vennila Mathivanan, Bethany A. Horsburgh, Katie Fisher, Orion Tong, Eunok Lee, Haelee Ahn, Ansari Shaik, Rémi Fromentin, Rebecca Hoh, Charline Bacchus-Souffan, Najla Nasr, Anthony L. Cunningham, Peter W. Hunt, Nicolas Chomont, Stuart G. Turville, Steven G. Deeks, Anthony D. Kelleher, Timothy E. Schlub, Sarah Palmer

**Affiliations:** 1 Centre for Virus Research, The Westmead Institute for Medical Research, Westmead, New South Wales, Australia.; 2 Faculty of Medicine and Health, The University of Sydney, Sydney, New South Wales, Australia.; 3 Post-graduation Program of Evolutionary Genetics and Molecular Biology, Federal University of São Carlos, São Carlos, Brazil.; 4 The Kirby Institute, University of New South Wales, Sydney, New South Wales, Australia.; 5 Division of Experimental Medicine, University of California, San Francisco, San Francisco, California, USA.; 6 Centre de Recherche du Centre Hospitalier de l’Université de Montréal, Montreal, Quebec, Canada.; 7 Department of Medicine, University of California, San Francisco, San Francisco, California, USA.; 8 Department of Microbiology, Infectiology and Immunology, Université de Montréal, Montreal, Quebec, Canada.

**Keywords:** AIDS/HIV, Infectious disease, Adaptive immunity, Molecular genetics, T cells

## Abstract

Despite long-term antiretroviral therapy (ART), HIV-1 persists within a reservoir of CD4^+^ T cells that contribute to viral rebound if treatment is interrupted. Identifying the cellular populations that contribute to the HIV-1 reservoir and understanding the mechanisms of viral persistence are necessary to achieve an effective cure. In this regard, through Full-Length Individual Proviral Sequencing, we observed that the HIV-1 proviral landscape was different and changed with time on ART across naive and memory CD4^+^ T cell subsets isolated from 24 participants. We found that the proportion of genetically intact HIV-1 proviruses was higher and persisted over time in effector memory CD4^+^ T cells when compared with naive, central, and transitional memory CD4^+^ T cells. Interestingly, we found that escape mutations remained stable over time within effector memory T cells during therapy. Finally, we provided evidence that Nef plays a role in the persistence of genetically intact HIV-1. These findings posit effector memory T cells as a key component of the HIV-1 reservoir and suggest Nef as an attractive therapeutic target.

## Graphical abstract

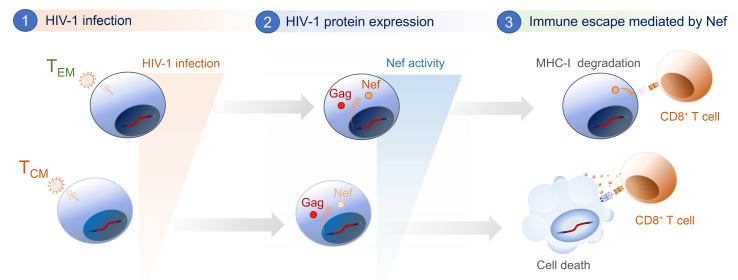

## Introduction

Genetic characterization of HIV-1 proviruses isolated from individuals on suppressive antiretroviral therapy (ART) has revealed that 2%–8% of persistent HIV-1 is genetically intact and potentially replication competent (1–6). Replication-competent proviruses are the main barrier to HIV-1 eradication, as they contribute to rebound in viral load if therapy is interrupted (7, 8). For this reason, determining the source of replication-competent HIV-1 is critical for identifying cellular targets for future curative strategies. Naive (Tn) and memory CD4^+^ T cells are a well-defined reservoir of replication-competent HIV-1, which is differentially distributed within different CD4^+^ T cell subsets (4–6, 9–11). To investigate the distribution of genetically intact and potentially replication-competent HIV-1 proviruses within Tn, central (Tcm), transitional (Ttm), and effector (Tem) memory CD4^+^ T cell subsets, our group developed the Full-Length Individual Proviral Sequencing (FLIPS) assay (4, 12). We found that genetically intact HIV-1 proviruses were unequally distributed between these CD4^+^ T cell subsets and identified Tem as an important source of replication-competent HIV-1 (4). Understanding the distribution of replication-competent HIV-1 between Tn and memory CD4^+^ T cell subsets and how replication-competent HIV-1 proviruses are maintained during long-term ART deserves further analysis.

In HIV-1^+^ individuals on ART, viral DNA levels remain stable after long-term effective treatment (13). However, the latent HIV-1 reservoir, as measured the by quantitative outgrowth assay, declines slowly, with a half-life of 44 years (14–16). Recent studies have shown that cellular proliferation and death contribute to changes in the genetic composition of persistent HIV-1 with time (10, 17–21). CD4^+^ T cell subsets exhibit unique qualities that likely influence the genetic landscape of persistent HIV-1 within them. For example, as cellular differentiation status increases (i.e., Tn < Tcm < Ttm < Tem), cells have higher rates of proliferation, HIV-1 gene expression, and susceptibility to cytotoxic T lymphocytes (CTLs), indicating that the processes that contribute to the dynamics of the HIV-1 reservoir over time likely affect the subsets differently (11, 17, 22–28). Therefore, characterization of the HIV-1 proviral landscape across the CD4^+^ T cell subsets requires further investigation.

In the face of CTL pressure, HIV-1 can evade CD8^+^ T cell recognition through mutations in major histocompatibility complex class I–restricted (MHC-I–restricted) epitopes (29, 30). Immune escape may also be mediated by the expression of viral proteins, such as negative effective factor (Nef), encoded by the *nef* gene. Nef is expressed early in the viral life cycle (31) and is crucial for viral pathogenesis and progression to AIDS in vivo (32). Nef can evade the innate and adaptive immune response through the downregulation of cell surface molecules, such as CD4 and MHC-I (33, 34). Downregulation of CD4 molecules from the cell surface allows efficient release of newly formed HIV-1 particles from infected cells (35), enhances virion infectivity (36), inhibits superinfection (37), and protects infected cells from antibody-dependent cellular cytotoxicity (38). Selective downregulation of HLA-A and HLA-B molecules by Nef allows HIV-1–infected cells to avoid CD8^+^ T cell recognition (39), while preventing killing by natural killer cells (34). Furthermore, Nef inhibits the activities of proapoptotic proteins to prevent programmed cell death of HIV-1–infected cells (40–42). However, the role of Nef in the persistence of genetically intact HIV-1 proviruses in individuals on long-term ART and its contribution to the HIV-1 proviral landscape across Tn and memory CD4^+^ T cell subsets have not been studied.

In this study, we provide evidence that the HIV-1 proviral landscape is different between Tn and memory CD4^+^ T cell subsets isolated from individuals on effective ART. Importantly, we found that Tem cells are a key component of the HIV-1 reservoir after long-term ART. Compared with other CD4^+^ T cell subsets, these cells harbor the highest frequency of genetically intact HIV-1 proviruses. We found that the persistence of genetically intact HIV-1 in Tem cells cannot be attributed to CTL escape mutations, which remain stable and do not increase over time during ART. Instead, we suggest that Nef contributes to the persistence of genetically intact HIV-1 and proviruses containing genes encoding immunogenic proteins such as Gag in Tem cells. This indicates that the presence of genetically intact *nef* supports the maintenance of the HIV-1 reservoir, and inhibition of Nef expression could play a role in reducing this reservoir.

## Results

### Genetically intact HIV-1 proviruses isolated from ART-suppressed individuals are enriched within Tem CD4^+^ T cells.

**Figure 1:**
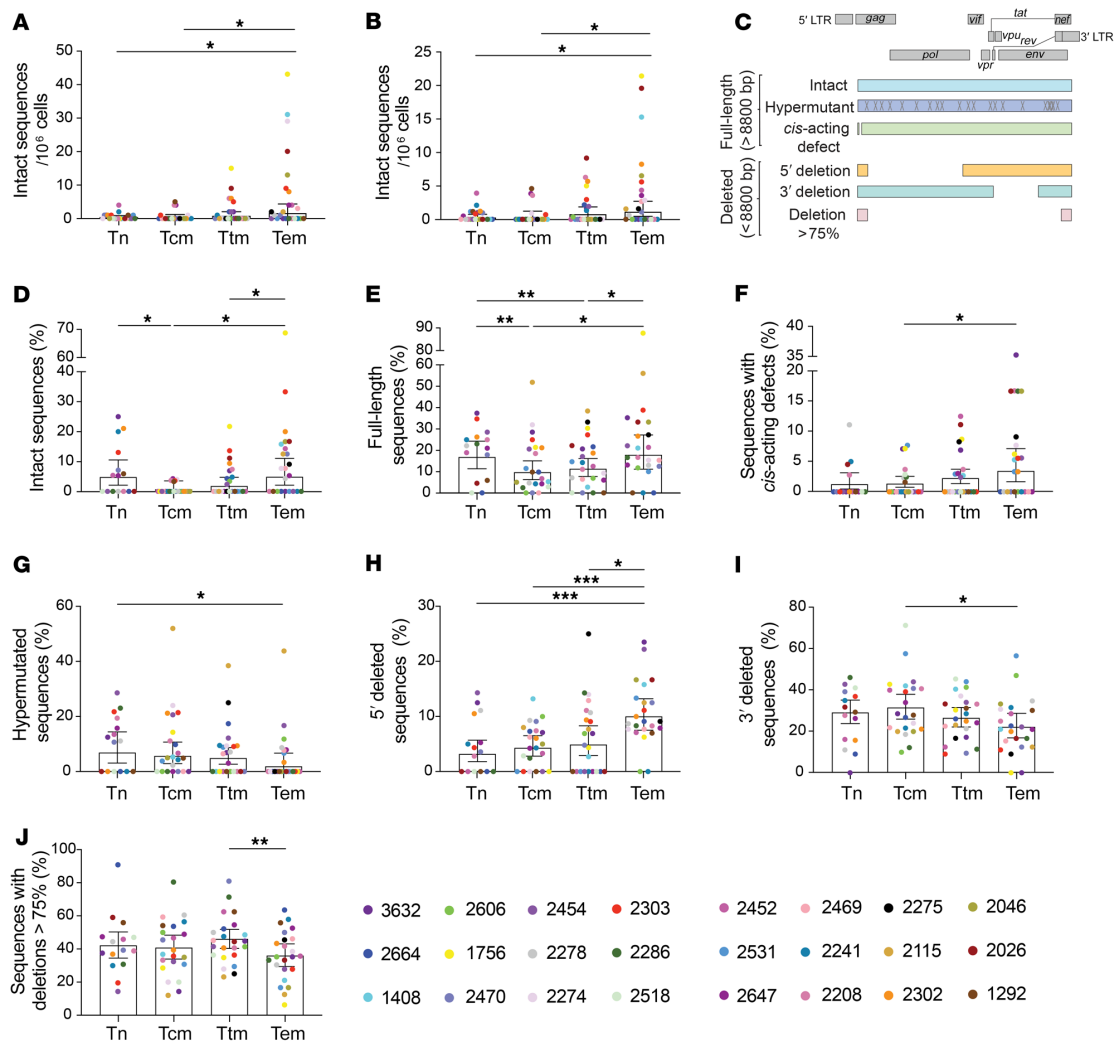
The HIV-1 proviral landscape is different between Tn and memory CD4^+^ T cell subsets isolated from ART-suppressed individuals. Near-full-length HIV-1 proviral sequences were obtained by FLIPS from Tn and memory CD4^+^ T cell subsets of participants on suppressive ART. Genetically intact HIV-1 proviral sequences were identified and the intact infection frequency was calculated using a mixed logistic model for each cell subset: (**A**) including all intact sequences and (**B**) counting identical intact proviral sequences only once for each cell subset. Data represent average genetically intact proviruses per 10^6^ cells ± 95% CIs. *P* values were calculated with a likelihood ratio test. (**C**) Proviral sequences were classified as full length (>8800 bp) or deleted (<8800 bp) and further categorized according to their predominant characteristic. In each cell subset, the percentage of each type of provirus was calculated: (**D**) intact; (**E**) full-length; (**F**) *cis*-acting defect; (**G**) hypermutated; (**H**) 5′ deleted; (**I**) 3′ deleted; and (**J**) 75% deleted. Genetically identical sequences were counted only once for each subset. Data represent the adjusted overall percentage ± 95% CI. *P* values were calculated with a likelihood ratio test. Tn, naive; Tcm, central memory; Ttm, transitional memory; Tem, effector memory. *P ≤ 0.05, **P ≤ 0.01, ***P ≤ 0.001.

Previously, we reported that genetically intact and potentially replication-competent HIV-1 proviruses are unequally distributed between Tn and memory CD4^+^ T cell subsets and proposed that Tem CD4^+^ T cells are an important component of the HIV-1 reservoir in 6 participants (4). Here, we further confirmed these findings by isolating Tn, Tcm, Ttm, and Tem CD4^+^ T cell subsets from the peripheral blood of 24 HIV-1^+^ ART-suppressed individuals (Supplemental Table 1; supplemental material available online with this article; https://doi.org/10.1172/JCI154422DS1) and analyzing near-full-length HIV-1 proviral sequences obtained by FLIPS. Genetically intact HIV-1 proviruses were identified as those sequences lacking *cis*-acting defects (packaging signal and major splice donor site [MSD] defects), internal deletions, hypermutation, frame shifts, or deleterious stop codons in essential open reading frames (ORFs). We identified 131 genetically intact HIV-1 proviruses (range 0–22 per participant) from a total of 2730 sequences (Supplemental Table 2). We calculated the intact HIV-1 infection frequency (intact HIV-1 proviruses per million cells) in each cell subset and observed the order of intact infection frequency from lowest to highest to be Tcm < Tn < Ttm < Tem (Figure 1A; ranging from 0.07 to 1.6 intact HIV-1 proviruses per million cells in Tcm and Tem cells, respectively). Importantly, we found evidence that Tem CD4^+^ T cells contained a higher infection frequency of genetically intact HIV-1 proviruses compared with the Tn and Tcm subsets (*P ≤* 0.05; Figure 1A), reconfirming our previous observations (4, 11).

HIV-1–infected CD4^+^ T cells can undergo antigen-, homeostatic-, or integration-site-driven proliferation, and proliferation rates vary between cell subsets (23, 28, 43, 44). As Tem cells exhibit higher rates of cellular proliferation than Tn and other memory cell subsets, we reasoned the high intact infection frequency observed in Tem cells may be due to proliferation of Tem cells harboring intact HIV-1 proviruses. To investigate this, we recalculated the intact infection frequency, this time counting identical sequences only once in each cell subset. We found no change in the order of intact infection frequency (Figure 1B; 0.07–1.1 intact HIV-1 proviruses per million cells in Tcm and Tem cells, respectively). Again, we found evidence that Tem cells contained a significantly higher infection frequency of genetically intact HIV-1 genomes per million cells than Tn and Tcm subsets (*P ≤* 0.05; Figure 1B). These results indicate that proliferation of cells containing genetically intact HIV-1 proviruses is not the only mechanism contributing to the persistence of intact HIV-1 in Tem cells.

### The HIV-1 proviral landscape is different between Tn and memory CD4^+^ T cell subsets.

As we had established that the distribution of genetically intact HIV-1 proviruses was different between the Tn and memory CD4^+^ T cell subsets, we further investigated how the HIV-1 proviral landscape is unique between these cells. Understanding how HIV-1 proviruses isolated from the different cell types differ at a sequence level may provide clues as to how genetically intact HIV-1 proviruses persist within the different subsets of CD4^+^ T cells. We classified HIV-1 proviruses according to their predominant genetic defect and compared the proportion of sequences within each classification between Tn and memory CD4^+^ T cell subsets (Figure 1C and Supplemental Table 3). HIV-1 proviruses were identified as full-length (>8800 bp) and sequentially classified as hypermutants, sequences with a *cis*-acting defect, or intact. To prevent any influence of cellular proliferation of HIV-1–infected cells on the calculated proportions, genetically identical HIV-1 proviral sequences were counted once for each cell subset. As shown in Figure 1, we found evidence that the proviral landscape is different between subsets. In addition to harboring a significantly higher proportion of genetically intact HIV-1 proviruses (*P* < 0.05; Figure 1D), Tem cells contained a substantially higher proportion of full-length proviruses than Tcm and Ttm cells (*P* < 0.05; Figure 1E), and a higher proportion of sequences with *cis-*acting defects compared with Tcm cells (*P* < 0.05; Figure 1F). Furthermore, the proportion of full-length HIV-1 proviruses within Tn cells was significantly higher than Tcm and Ttm cells (*P* < 0.01; Figure 1E), and a higher proportion of hypermutated sequences was found in Tn cells compared with Tem cells (*P* < 0.05; Figure 1G).

In addition, because a large proportion of HIV-1 proviruses isolated from individuals on ART harbor internal deletions (1, 2, 4), we classified HIV-1 proviruses based on the size and deletion position as follows: deletions of more than 75% of genome length, a predominantly 5′ deletion, or a predominantly 3′ deletion. Counting identical sequences once, we found that Tem cells contained a significantly higher proportion of proviruses with a 5′ deletion than Tn, Tcm, and Ttm cells (*P* < 0.05; Figure 1H). In contrast, Tem cells contained a significantly lower proportion of proviruses with a 3′ deletion and a deletion of more than 75% of the genome length than Tcm and Ttm cells, respectively (*P* < 0.05 and *P* < 0.01, respectively; Figure 1, I and J). Taken together, these results show that the proviral landscape is different between Tn and memory CD4^+^ T cell subsets.

### The distribution of full-length HIV-1 proviruses within memory CD4^+^ T cells is similar after 1 round of viral replication.

**Figure 2:**
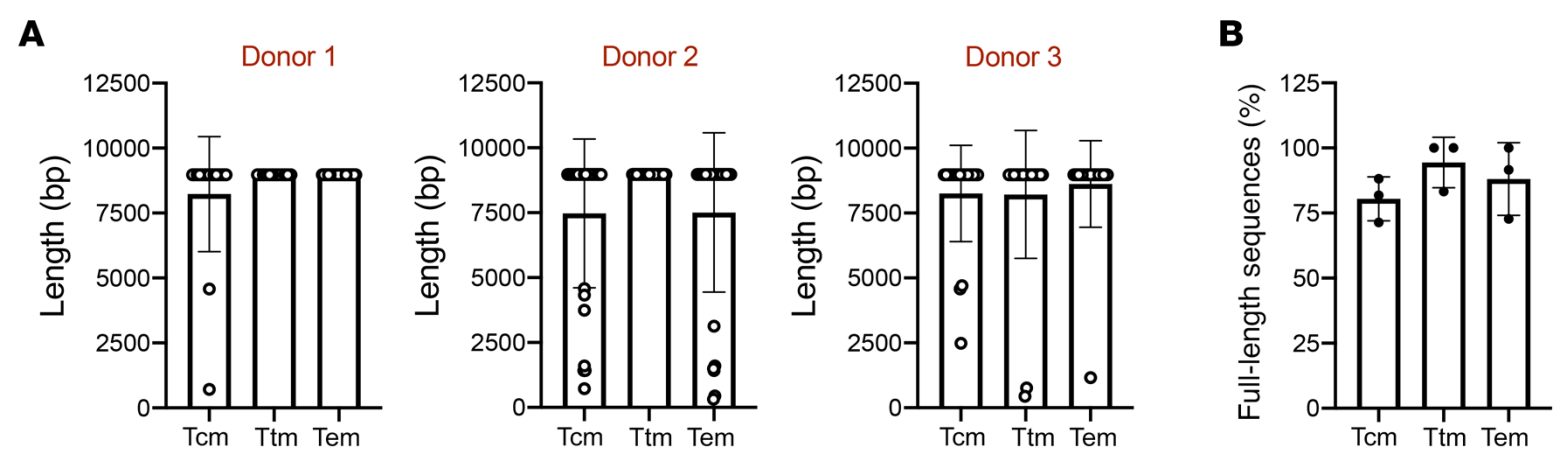
The HIV-1 proviral landscape of memory CD4^+^ T cell subsets is similar after 1 round of viral replication. Memory CD4^+^ T cells obtained from 3 HIV-1^–^ donors were infected in vitro with HIV-1, and proviral sequences were obtained by FLIPS. (**A**) HIV-1 proviral sequence length in CD4^+^ T cell memory subsets. Each data point represents a single proviral sequence. (**B**) The percentage of full-length sequences in each CD4^+^ T cell subset is shown. Each data point represents a single donor. Data represent the mean ± SD. Statistical significance was determined by Kruskal-Wallis followed by Dunn’s post test. Tcm, central memory; Ttm, transitional memory; Tem, effector memory.

We observed a higher proportion of intact and full-length proviral sequences in Tem subsets compared with Ttm and Tcm subsets (Figure 1, D and E). As Tem cells have been shown to have a higher susceptibility to HIV-1 infection, due to a higher metabolic rate improving the early steps of the viral replication, which includes reverse transcription (45), this might explain the higher proportion of full-length HIV-1 proviruses observed in this subset. To evaluate whether the unequal proportion of full-length proviruses across the CD4^+^ T cell subsets can be observed at early stages of infection, we sorted memory Tcm, Ttm, and Tem CD4^+^ T cells from 3 HIV-1^–^ donors (Supplemental Figure 1) and infected them for 20 hours with the HIV-1 laboratory strain, NL4-3. After 20 hours of infection, equivalent to 1 replication cycle (46), we performed FLIPS and quantified the proportion of full-length sequences present in each CD4^+^ T cell subset (Figure 2A). As shown in Figure 2B, no difference in the proportion of full-length proviruses was observed between the different subsets of CD4^+^ T cells (Tcm/Ttm, *P* = 0.28; Tcm/Tem, *P* = 0.86; Ttm/Tem, *P* > 0.99). This suggests that the proviral landscape in HIV-1^+^ individuals on ART is not driven by differences in early stages of viral replication.

### The HIV-1 proviral landscape may change over time within Tn and memory CD4^+^ T cell subsets during ART.

**Figure 3:**
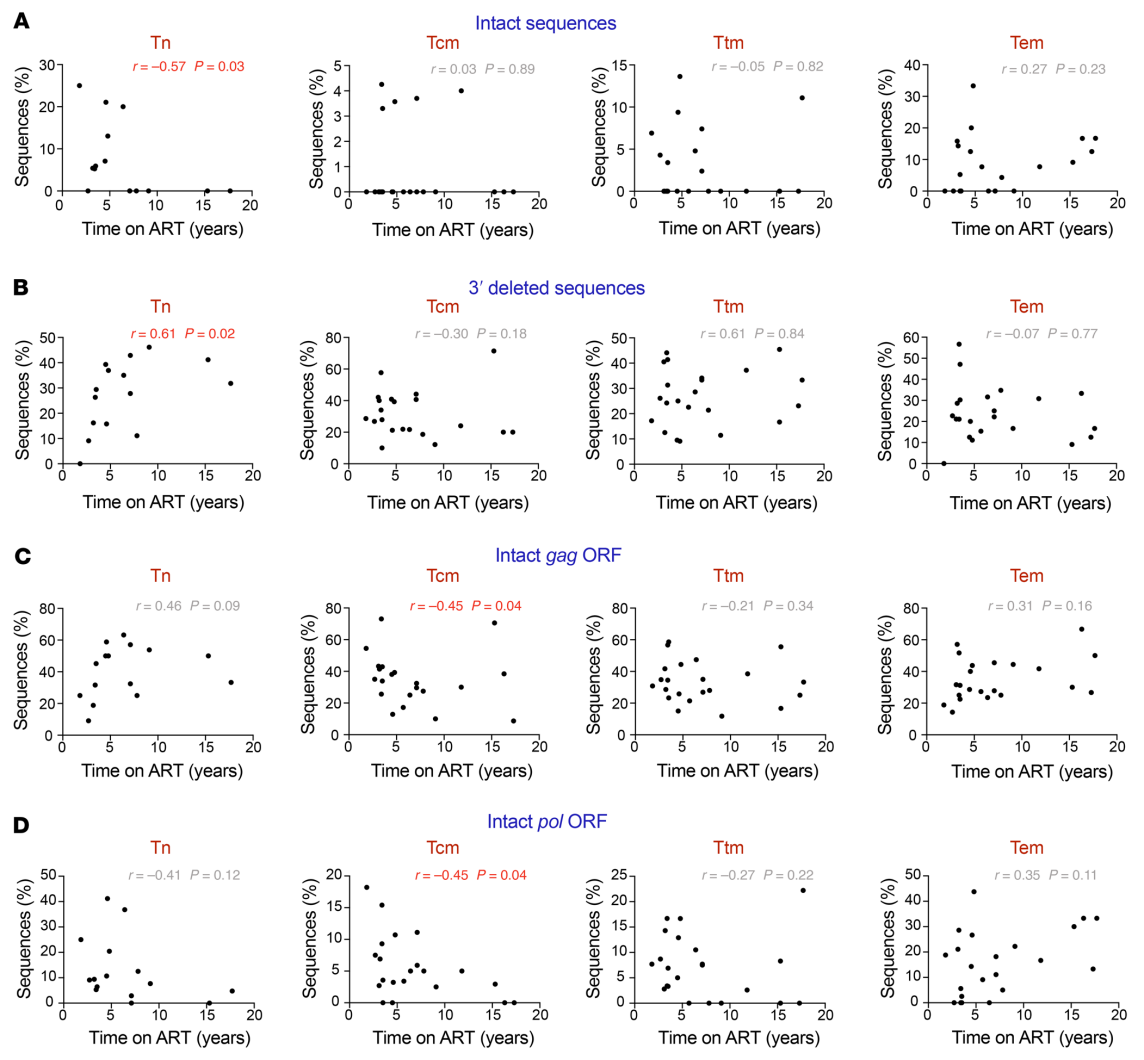
The HIV-1 proviral landscape changes over time in Tn and memory CD4^+^ T cell subsets. Correlation analysis of the relationship between the percentage of (**A**) intact HIV-1 proviral sequences; (**B**) 3′ deleted HIV-1 proviral sequences; (**C**) HIV-1 proviral sequences with an intact *gag* ORF; and (**D**) HIV-1 proviral sequences with an intact *pol* ORF within each participant and time on ART (years) across Tn, Tcm, Ttm, and Tem cells. Each data point represents the percentage of sequences obtained per participant. Statistical significance was calculated by Spearman’s correlation test. *P ≤* 0.05 values are shown in red. Tn, naive; Tcm, central memory; Ttm, transitional memory; Tem, effector memory; ORF, open reading frame.

The absence of a difference among the Tcm, Ttm, and Tem cell proviral landscapes following 1 round of infection indicates that the HIV-1 proviral landscape observed in individuals on ART may be shaped over time. Multiple studies have shown that the HIV-1 reservoir is dynamic, with genetically intact HIV-1 proviruses isolated from total CD4^+^ T cells decreasing over time (17–21, 47, 48). As the 24 participants included in this study were on ART for different lengths of time (median, 5.2 years; range 1.8–17.7 years; Supplemental Table 1), we conducted an interparticipant cross-sectional analysis. This allowed us to determine if there was a correlation between the proportion of HIV-1 proviruses categorized in Figure 1C and ART duration (Supplemental Table 3). To prevent any influence of cellular proliferation, in conducting these analyses we counted identical sequences only once for each subset. First, we looked at the relationship between the proportion of genetically intact HIV-1 proviruses and ART duration in each cell subset. We found that the proportion of intact proviruses in Tn cells correlates negatively with ART duration (Spearman’s *r* = –0.57, *P* = 0.03; Figure 3A), suggesting that the proportion of intact proviruses may decrease with time on ART in Tn cells. However, no significant correlation between the proportion of intact proviruses and ART duration for the other cell subsets was observed. We next found a positive correlation in Tn cells between the proportion of proviruses with a 3′ deletion and ART duration (Spearman’s *r* = 0.61, *P* = 0.02; Figure 3B). We did not observe any significant association between ART duration and the other categories of defective proviruses (full-length, *cis-*acting defect, hypermutant, 5′ deletion, or deletion of more than 75%; Supplemental Table 4). Interestingly, in Tem cells, correlation analyses indicated a relationship between the proportion of full-length (>8800 bp) proviruses and ART duration (Spearman’s *r* = 0.38, *P* = 0.08; Supplemental Figure 2A). As these data were normally distributed, we conducted linear regression analyses and found that in Tem cells the proportion of full-length proviruses correlated positively with ART duration (slope = 1.46 [95% CI = 0.41–2.5], *P* = 0.009; Supplemental Figure 2B). Interestingly, these findings suggest that within Tem cells full-length HIV-1 proviruses may be positively selected in participants on long-term ART.

We next investigated whether the HIV-1 genes *gag*, *pol*, and *nef*, which are reported to encode the most immunogenic viral proteins (49, 50), are differentially selected within Tn and memory CD4^+^ T cell subsets, as this may provide more information as to the selection pressures on these cells. We identified intact ORFs for *gag*, *pol*, and *nef* within each proviral sequence (Supplemental Table 3). ORFs were identified as intact if they had a start codon and lacked internal deletions, frame shifts, or premature stop codons. To prevent any influence of cellular proliferation, identical proviral sequences were counted only once for each cell subset. Correlation analyses were performed to identify the relationships between the proportion of each intact ORF and ART duration (Figure 3, C and D, and Supplemental Figure 2C). In the Tcm cell subset, we found a negative correlation between the proportion of intact *gag* ORFs and ART duration (Spearman’s *r* = –0.45, *P* = 0.04; Figure 3C) as well as the proportion of intact *pol* ORFs and ART duration (Spearman’s *r* = –0.45, *P* = 0.04; Figure 3D), suggesting that these ORFs may be negatively selected or reduced over time in Tcm cells. In contrast, we did not find evidence for a relationship between the proportion of *gag*, *pol*, or *nef* and ART duration in the other cell subsets (Figure 3, C and D, and Supplemental Figure 2C).

### The proportion of sequences harboring recognizable CTL epitopes negatively correlates with time on ART in Tcm CD4^+^ T cells.

**Figure 4:**
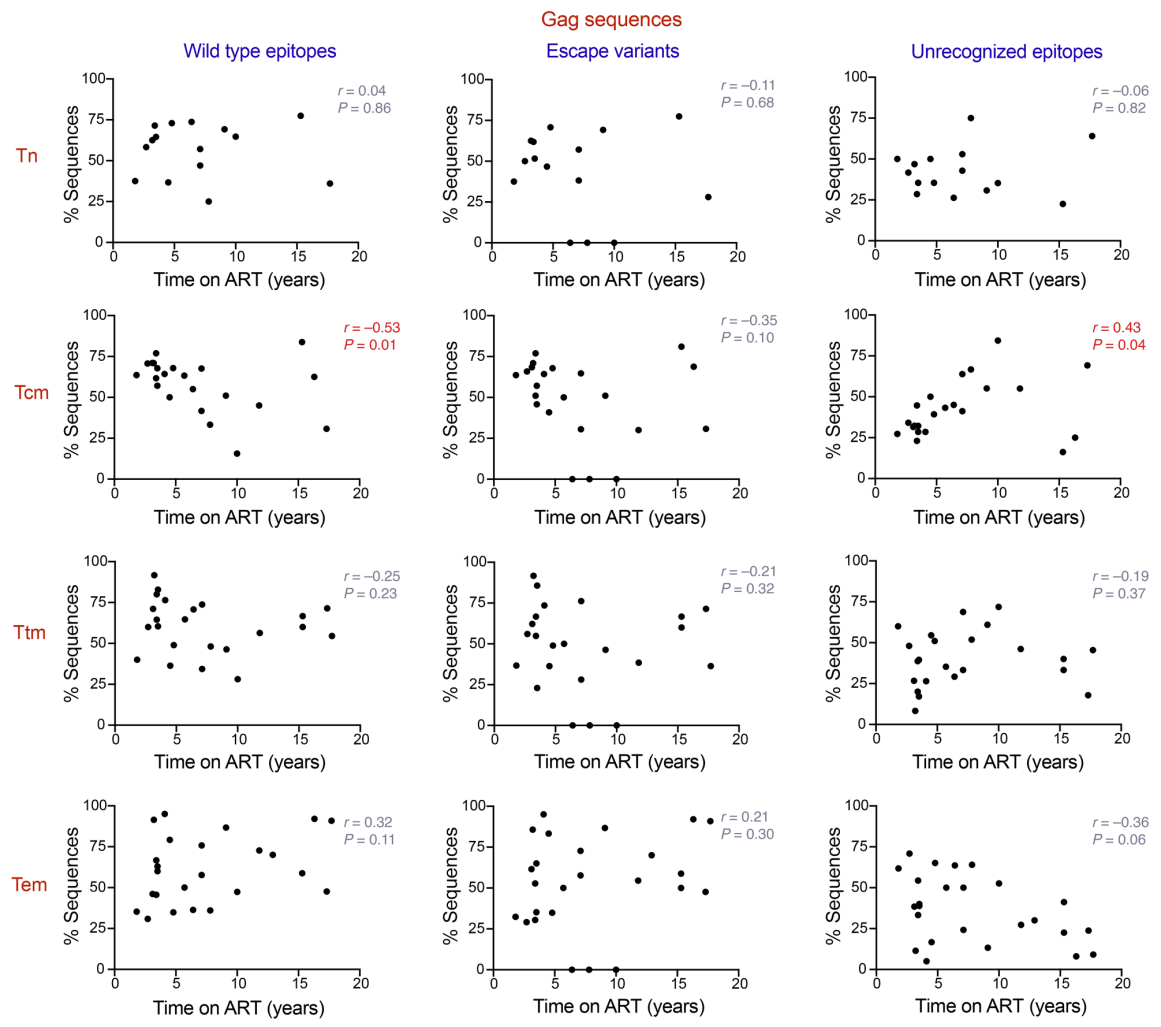
Proportion of HIV-1 proviral sequences expressing CTL WT and unrecognizable epitopes for Gag correlates with time on ART in Tcm CD4^+^ T cells. Correlation analysis of the relationship between the percentage of HIV-1 proviral sequences harboring WT epitopes, escape variants, and unrecognizable epitopes and time on ART (years) for the viral proteins Gag, across Tn, Tcm, Ttm, and Tem cells. Each data point represents the percentage of sequences obtained per participant. Statistical significance was calculated by Spearman’s correlation test. *P ≤* 0.05 values are shown in red. Tn, naive; Tcm, central memory; Ttm, transitional memory; Tem, effector memory.

**Figure 5:**
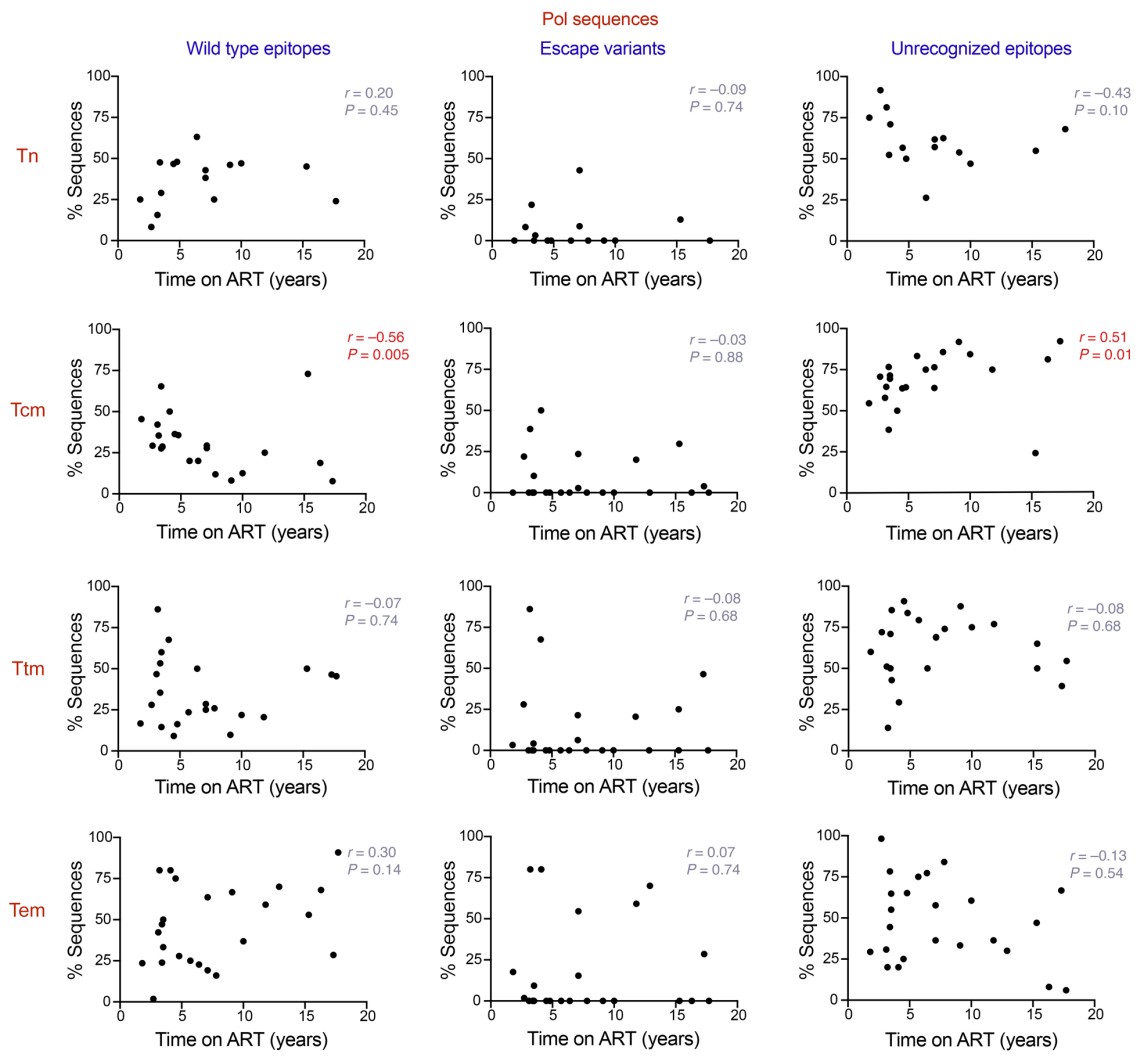
Proportion of HIV-1 proviral sequences expressing CTL WT and unrecognizable epitopes for Pol correlates with time on ART in Tcm CD4^+^ T cells. Correlation analysis of the relationship between the percentage of HIV-1 proviral sequences harboring WT epitopes, escape variants, and unrecognizable epitopes and time on ART (years) for the viral proteins Pol, across Tn, Tcm, Ttm, and Tem cells. Each data point represents the percentage of sequences obtained per participant. Statistical significance was calculated by Spearman’s correlation test. *P ≤* 0.05 values are shown in red. Tn, naive; Tcm, central memory; Ttm, transitional memory; Tem, effector memory.

Our results, along with those of previous studies, suggest that the HIV-1 proviral landscape can be shaped by immune pressure exerted by the activity of CTLs (18). Unless treatment is initiated during acute infection, the HIV-1 reservoir becomes dominated by viral variants containing CTL escape mutations that are resistant to the immune response (30). In addition, as demonstrated by Pollack and collaborators, some classes of defective proviruses can express HIV-1 epitopes and be recognized by CTLs (17). Because we observed that the proportion of sequences containing genetically intact *gag* and *pol* ORFs correlates negatively with time on ART in Tcm cells, we quantified the proportion of sequences harboring WT CTL epitopes, CTL escape variants, and unrecognizable epitopes within each cell subset for Gag and Pol amino acid sequences. The epitopes were classified as unrecognizable if they lacked a start codon and/or contained premature stop codons and/or frame shift mutations. In addition, each epitope was considered in the analysis only if the HLA type of the corresponding participant was coincident with the HLA restriction for the identified epitope. The same analysis was performed for Nef sequences, because Nef has been reported as one of the most immunogenic HIV-1 proteins, along with Gag and Pol (49, 50). We quantified the proportion of WT epitopes, escape variants, and unrecognizable epitopes across the Tn and memory CD4^+^ T cell subsets for Gag, Pol, and Nef sequences (Supplemental Figure 3). Our analysis showed a significantly higher proportion of Pol sequences with unrecognizable epitopes in Tcm cells when compared with Tem cells (*P* < 0.05; Supplemental Figure 3B). In contrast, we did not observe any significant differences when the other ORFs and categories of epitopes were compared between the different CD4^+^ T cell subsets. However, when we analyzed the proportion of sequences harboring WT epitopes, escape variants, and unrecognizable epitopes in an interparticipant cross-sectional analysis, we observed that the proportion of sequences containing Gag and Pol WT epitopes negatively correlates with time on ART in Tcm cells (Gag, Spearman’s *r* = –0.53, *P* = 0.01, Figure 4; Pol, Spearman’s *r* = –0.56, *P* = 0.005; Figure 5). Surprisingly, we did not see a correlation between the proportion of escape variants and time on ART for any of the cell subsets. However, we found that unrecognizable epitopes showed a positive correlation for Gag and Pol with time on ART in the Tcm subset (Gag, Spearman’s *r* = 0.43, *P* = 0.04; Figure 4; Pol, Spearman’s *r* = 0.51, *P* = 0.01; Figure 5). This result, in addition to the negative association between the proportion of WT epitopes and ART duration for Gag and Pol in Tcm cells, suggests that the decrease of sequences with intact *gag* and *pol* genomic regions in Tcm cells, may be a consequence of CTL activity exerted over time. For Nef, we did not observe a correlation between time and WT epitopes, escape variants, and unrecognizable epitopes across Tn and memory CD4^+^ T cells (Supplemental Figure 4).

### The proportion of HIV-1 proviral sequences with intact nef ORFs is higher in Tem cells.

**Figure 6:**
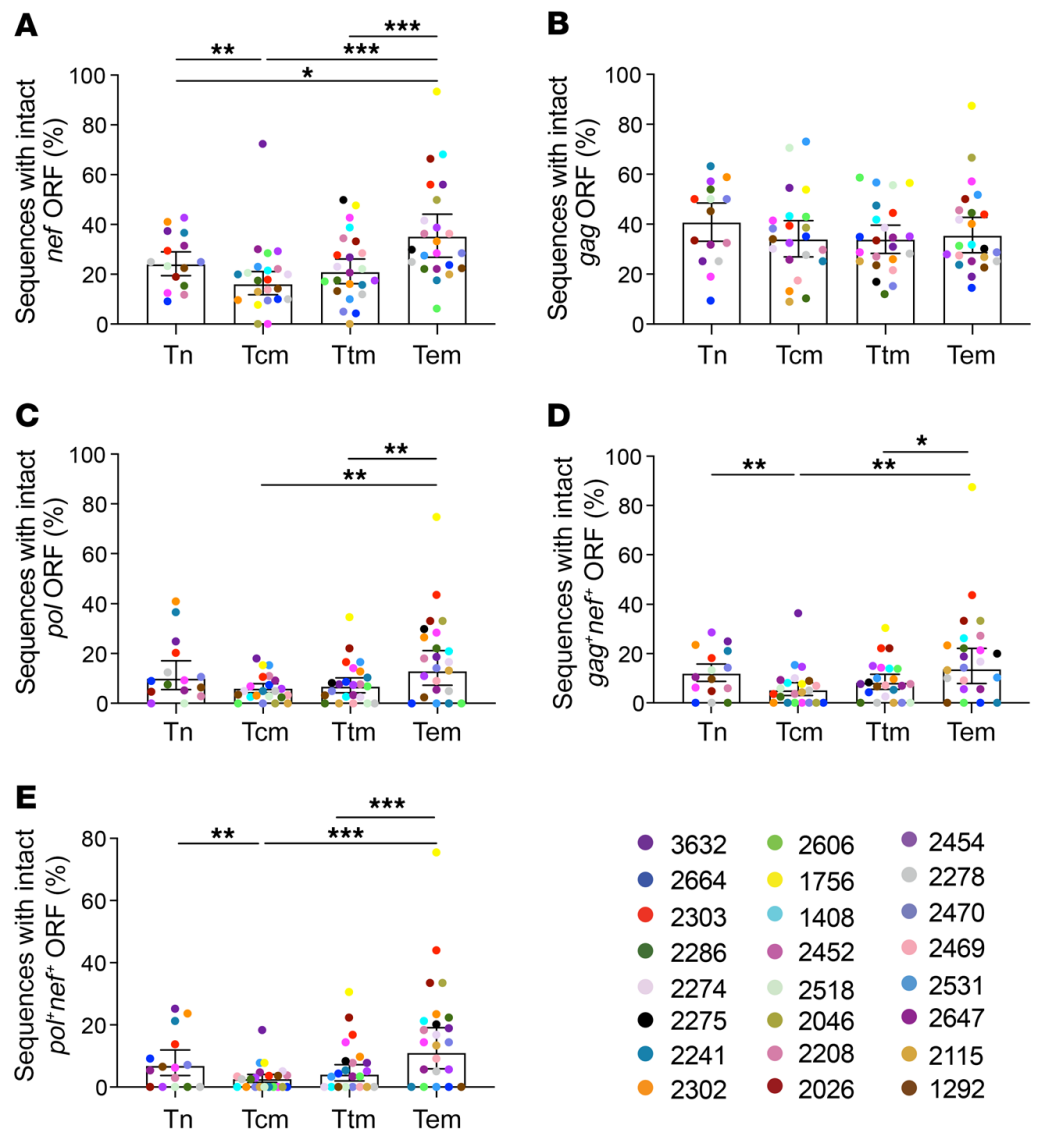
The proportion of HIV-1 proviral sequences with intact *nef* ORFs is higher in Tem cells. The percentage of sequences carrying genetically intact ORFs for (**A**) *nef;* (**B**) *gag*; and (**C**) *pol* was quantified across Tn and memory CD4^+^ T cells using a mixed logistic model. The percentage of HIV-1 proviral sequences harboring genetically intact *gag* (**D**) or *pol* (**E**) in combination with genetically intact *nef* within Tn and memory CD4^+^ T cells is shown. Data represent the adjusted overall percentage ± 95% CI. *P* values were calculated with a likelihood ratio test. Tn, naive; Tcm, central memory; Ttm, transitional memory; Tem, effector memory; ORF, open reading frame. *P ≤ 0.05, **P ≤ 0.01, ***P ≤ 0.001.

Because we observed that the proportion of sequences containing intact *gag* and *pol* ORFs decrease in Tcm cells, and we did not observe an enrichment of CTL escape variants or unrecognized epitopes in Gag and Pol sequences in Tem cells over time on ART, we next aimed to investigate how Tem cells are protected from CTL elimination. Notably, when we compared the proviral landscape between Tn and memory CD4^+^ T cell subsets, we found that Tem cells harbor a higher proportion of proviruses with a 5′ deletion compared with all other subsets (Figure 1H). We hypothesized that the viral protein Nef, whose ORF sits within the 3′ end of the HIV-1 genome, may protect HIV-1–infected Tem cells from immune pressure. To investigate this idea, first we quantified the proportion of sequences expressing an intact *nef* ORF in each cell subset and found that Tem cells had a significantly higher proportion of intact *nef* ORFs than all the other subsets (*P* < 0.05; Figure 6A). We next compared the proportion of sequences between the cell subsets containing an intact *gag* or *pol* ORF. We found no difference in the proportion of sequences with an intact *gag* ORF between the cell subsets (Figure 6B). However, we found that Tem cells contained a higher proportion of sequences with an intact *pol* ORF compared with the other memory subsets (*P* < 0.01; Figure 6C). Interestingly, when we quantified the proportion of sequences containing genetically intact *gag* in combination with intact *nef*, we found a higher proportion of cells with intact *gag* and *nef* ORFs (*gag*^+^*nef*^+^) in Tem cells compared with Tcm and Ttm cells (*P* < 0.05; Figure 6D). Similarly, we observed the proportion of sequences with genetically intact *pol* and *nef* (*pol*^+^*nef*^+^) was higher in Tem cells compared with the other memory T cell subsets (*P* < 0.001; Figure 6E).

### Defective HIV-1 proviruses harboring large internal deletions produce Nef.

**Figure 7:**
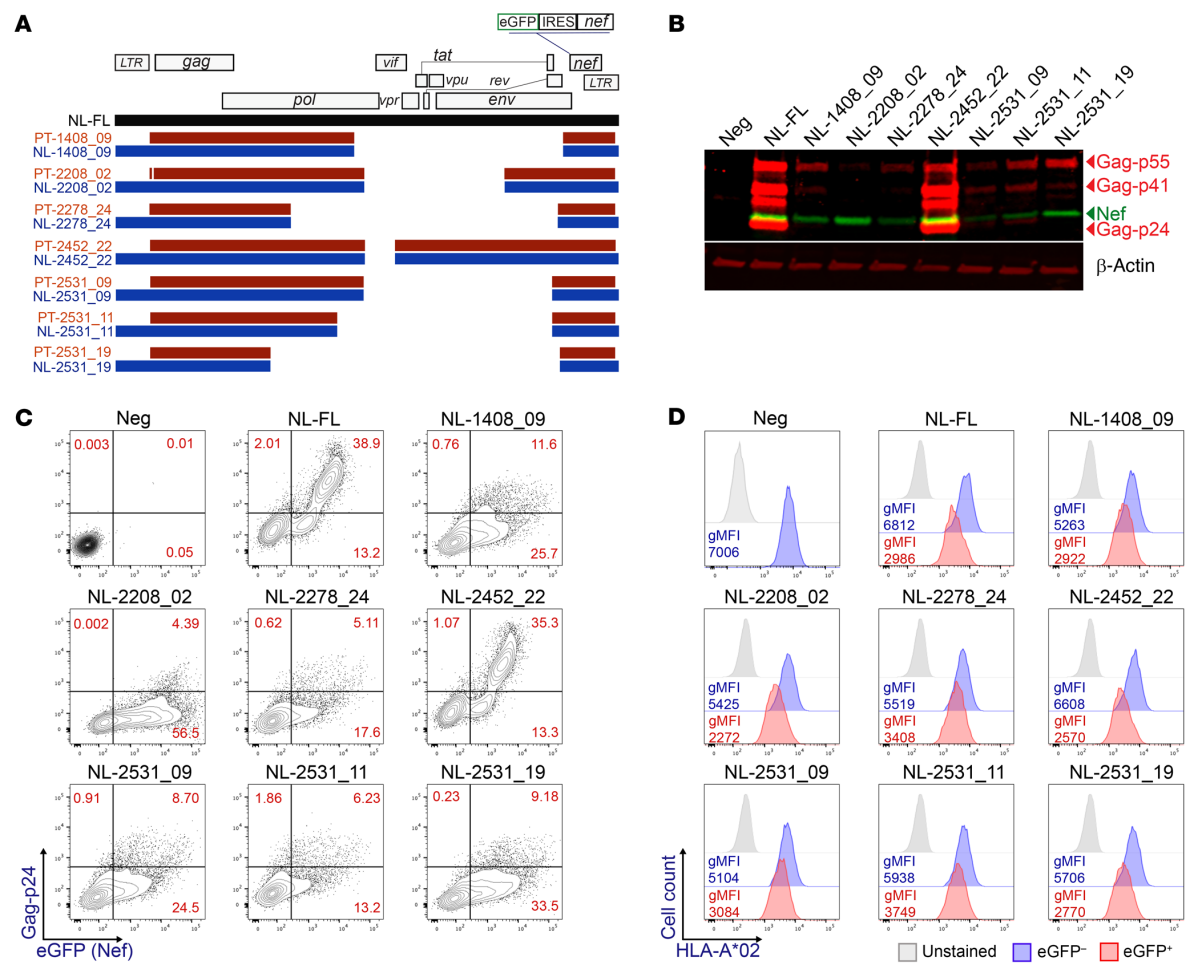
Defective proviruses can express Gag and functional Nef. (**A**) Schematic representing deletions in HIV-1 proviral sequences obtained from Tem CD4^+^ T cells from HIV-1^+^ participants (brown) and deletions generated in NL4-3-eGFP constructs (blue). The full-length HIV-1 NL4-3 construct is represented in black (NL-FL). HEK293T cells were transfected with HIV-1 NL constructs. (**B**) Representative Western blot of 2 independent experiments showing Nef (green) and Gag (top-red) expression. β-Actin was used as a protein loading control (bottom-red). (**C**) Representative flow cytometry of 2 independent experiments showing eGFP and p24 expression in transfected HEK293T cells. (**D**) Representative histograms of 2 independent experiments showing HLA-A*02 gMFI in eGFP^–^ and eGFP^+^ cells.

As most of the sequences analyzed were genetically defective, due to large internal deletions, it was uncertain whether defective proviruses harboring intact *gag* or *nef* genomic regions could produce these HIV-1 proteins. To investigate this question, we selected 7 proviral sequences from CD4^+^ Tem cells of 5 participants that contained large internal deletions but genetically intact *gag* and *nef* ORFs (Supplemental Table 5). We reproduced the deletion of each participant proviral sequence within an HIV-1 NL4-3 construct (Figure 7A). To quantify the expression of Nef, the gene for a green fluorescent protein (eGFP) was introduced into the *nef* ORF (Figure 7A). We transfected HEK293T cells with these constructs and used a vector containing the full-length HIV-1 NL4-3 sequence as a positive control. At day 2 after transfection, Gag and Nef protein expression was assessed by Western blot (Figure 7B) and quantified by flow cytometry, using eGFP as an indicator of Nef expression (Figure 7C and Supplemental Figure 5A). Interestingly, all the proviral constructs expressed Gag and Nef, but at different levels, with Nef expressed more efficiently than Gag (Figure 7C). Finally, to evaluate whether these defective proviruses produced functional Nef, cell surface expression of MHC-I (HLA-A*02) was quantified. We observed downregulation of MHC-I on the surface of the eGFP^+^ cells (Figure 7D and Supplemental Figure 5B), indicating that these defective proviruses express functional Nef.

### The proportion of gag^+^nef^+^ and pol^+^nef^+^ sequences increases after CTL clearance ex vivo.

**Figure 8:**
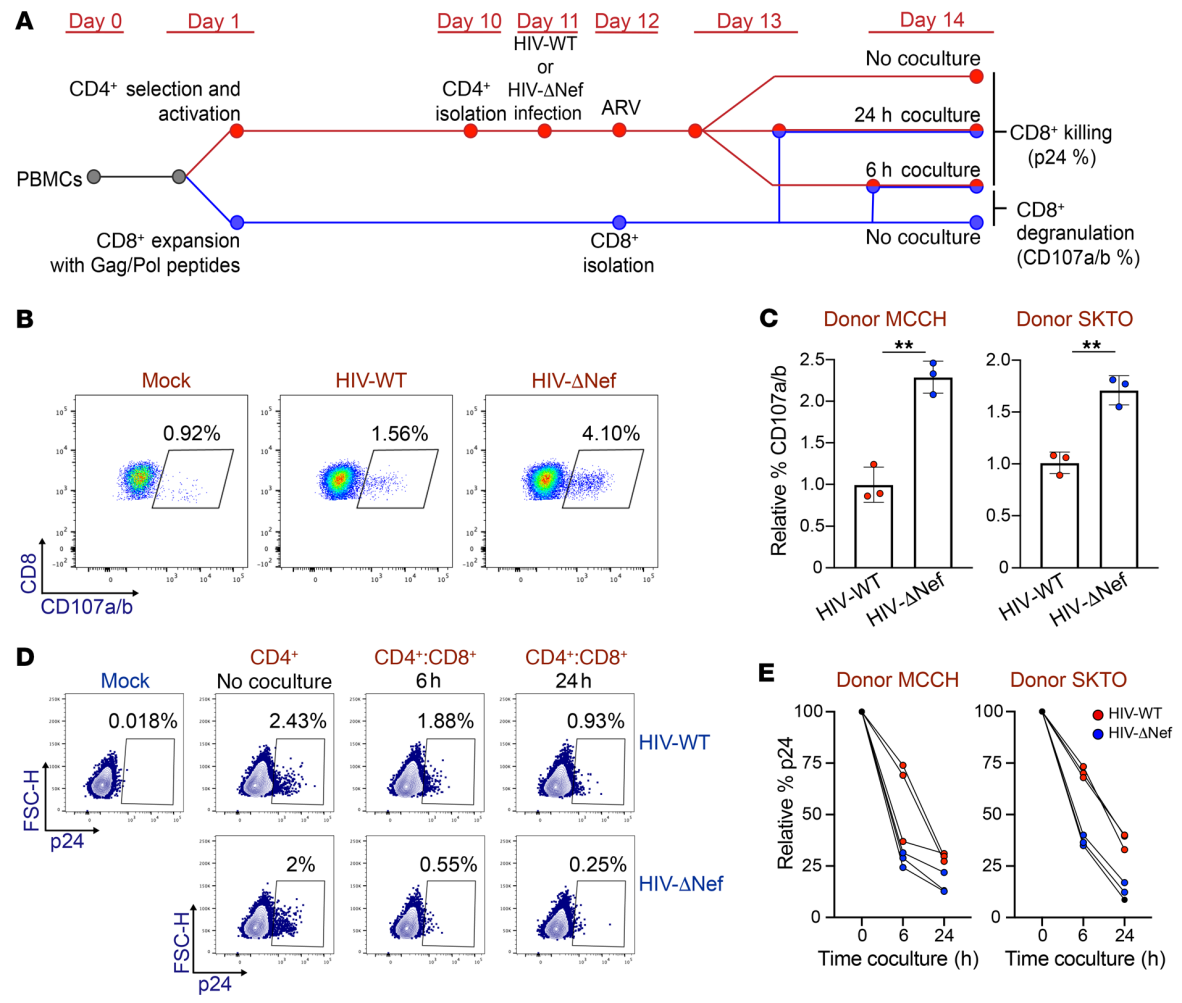
Nef^–^ HIV-1 induces higher CD8^+^ T cell activity. CD4^+^ T cells isolated from HIV-1^+^ donors were infected with HIV-WT or HIV-ΔNef and cocultured with autologous CD8^+^ T cells. (**A**) Timeline illustrating the experimental procedure, as described in Methods. (**B**) Flow cytometry of CD107a/b expression in CD8^+^ T cells from a representative experiment. (**C**) Expression of CD107a/b relative to values obtained from CD8^+^ T cells cocultured with HIV-WT–infected cells. Data represent mean ± SD. Statistical significance was determined by 2-tailed Student’s *t* test. Each experiment was performed in triplicate. **P ≤ 0.01. (**D**) Representative flow cytometry of 2 independent experiments showing p24 expression of cells infected with HIV-WT or HIV-ΔNef with or without coculture with autologous CD8^+^ T cells. (**E**) p24 values of cells infected with HIV-WT or HIV-ΔNef after 6 and 24 hours of coculture with CD8^+^ T cells relative to p24 expression in the CD4^+^ T cell–only condition.

To further investigate the role of Nef in protecting HIV-1–infected cells from immune pressure, we next used an ex vivo model to investigate whether the presence of *nef* protects sequences containing *gag* and *pol* from CD8^+^ T cell clearance. We purified CD4^+^ T cells from 3 HIV-1^+^ participants on ART (Supplemental Table 6) and infected them with the laboratory strain HIV-1 NL4-3 (HIV-WT) or an HIV-1 NL4-3 strain with a deletion of the *nef* ORF (HIV-ΔNef) (Figure 8A). In parallel, we expanded HIV-1–specific CD8^+^ T cells with a pool of peptides from Gag and Pol. At day 2 after infection, we cocultured the CD4^+^ T cells infected with HIV-WT or HIV-ΔNef with the expanded CD8^+^ T cells. Next, we compared the ability of both viruses to induce effective CTL antiviral activity by measuring the expression of CD107a/b (a marker for degranulation) and determining the killing efficiency of HIV-1–infected CD4^+^ T cells (Figure 8, B–E). As expected, cells infected with HIV-ΔNef induced a higher level of degranulation of the expanded CD8^+^ T cells (*P* < 0.05; Figure 8, B and C). In addition, the absence of *nef* in HIV-1–infected cells promoted a faster decline in the proportion of HIV-1^+^ cells in the CD4/CD8 coculture, indicating that CD8^+^ T cells can kill cells infected with HIV-ΔNef (Figure 8, D and E) more efficiently. These results validated our model, allowing us to test whether Nef can protect cells expressing immunogenic proteins such as Gag and Pol and to evaluate whether the presence of the *nef* ORF can affect the HIV-1 proviral landscape.

**Figure 9:**
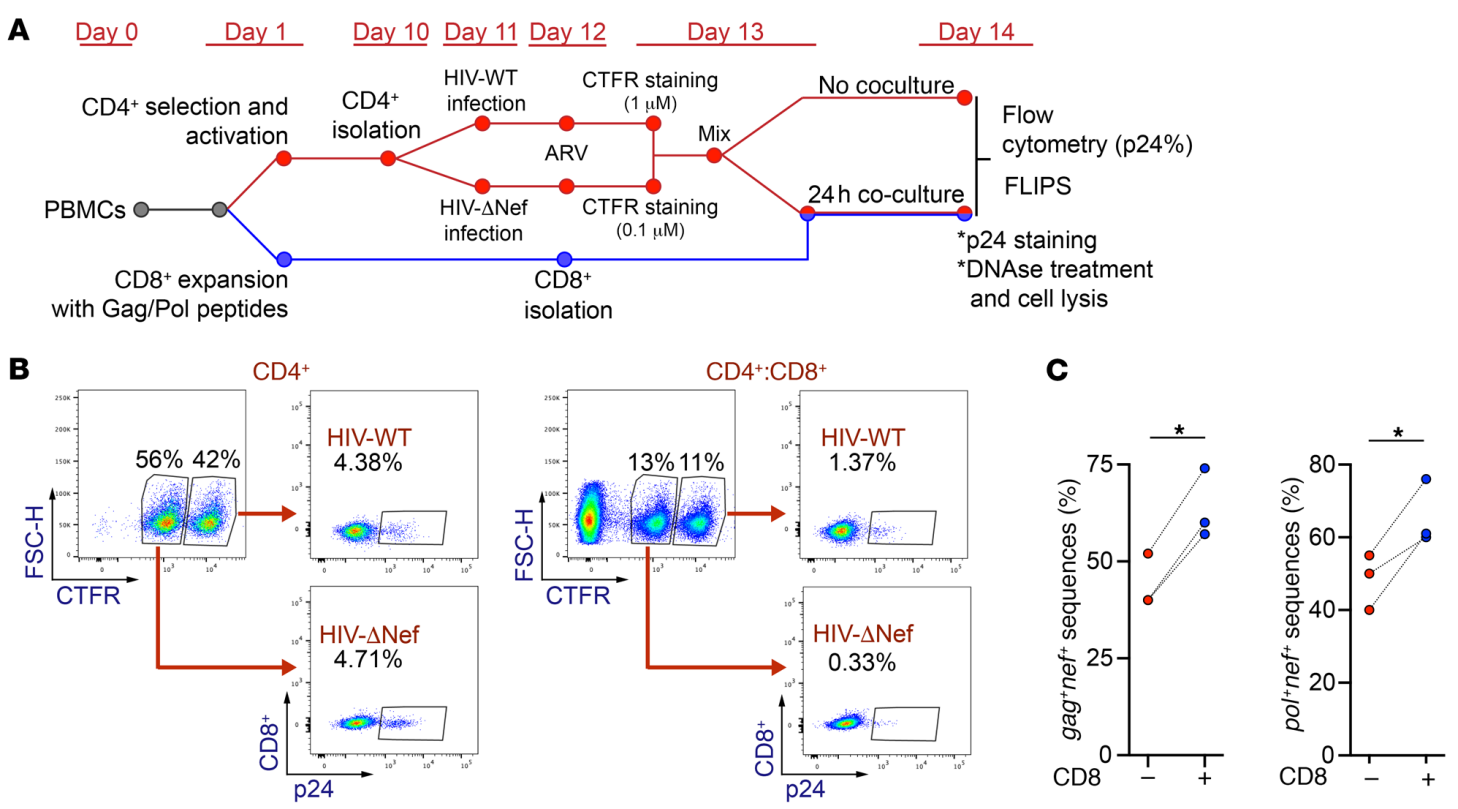
The proportion of HIV-1 proviruses containing *nef* increases after CD8^+^ T cell clearance. (**A**) Timeline illustrating the experimental procedure for the viral competition assay, as described in Methods. (**B**) Representative flow cytometry of 3 independent experiments showing p24 values from cells infected with HIV-WT or HIV-ΔNef after the coculture with CD8^+^ T cells. (**C**) Proportion of HIV-1 sequences containing *gag*^+^*nef*^–^ or *pol*^+^*nef*^+^ in infected cells in the presence and absence of CD8^+^ T cell coculture. Results obtained from 3 independent donors are shown. Statistical significance was determined by paired 2-tailed *t* test. *P ≤ 0.05.

To investigate if the presence of intact *nef* can influence the proviral landscape, and to determine whether the proportion of proviral sequences containing genetically intact *gag^+^nef^+^* or *pol^+^nef^+^* can be positively selected by the action of HIV-1–specific CD8^+^ T cells, we performed a viral competition assay (Figure 9A). This assay allowed us to quantify the ratio of HIV-WT– to HIV-ΔNef–infected cells after exposure to the autologous CD8^+^ T cells expanded by the Gag and Pol peptides. As expected, CD8^+^ T cells efficiently killed more cells infected with HIV-ΔNef than cells infected with HIV-WT in our viral competition assay (Figure 9B). Next, to quantify the proportion of HIV-1 sequences containing intact *gag^+^nef^+^* and *pol^+^nef^+^*, we performed FLIPS after the coculture with the expanded CD8^+^ T cells. As a control, infected CD4^+^ T cells that were not cocultured with CD8^+^ T cells were sequenced to determine the basal proportion of both proviruses. Interestingly, after the coculture with the expanded CD8^+^ T cells, the proportion of proviruses containing intact *gag*^+^*nef^+^* (*P* < 0.05; Figure 9C) and *pol^+^nef^+^* (*P* < 0.05; Figure 9C) increased significantly. These results indicate that the presence of intact *nef* affects the HIV-1 proviral landscape, protecting HIV-1–infected CD4^+^ T cells from CTL clearance.

### Nef activity is higher in HIV-1–infected Tem CD4^+^ T cells.

**Figure 10:**
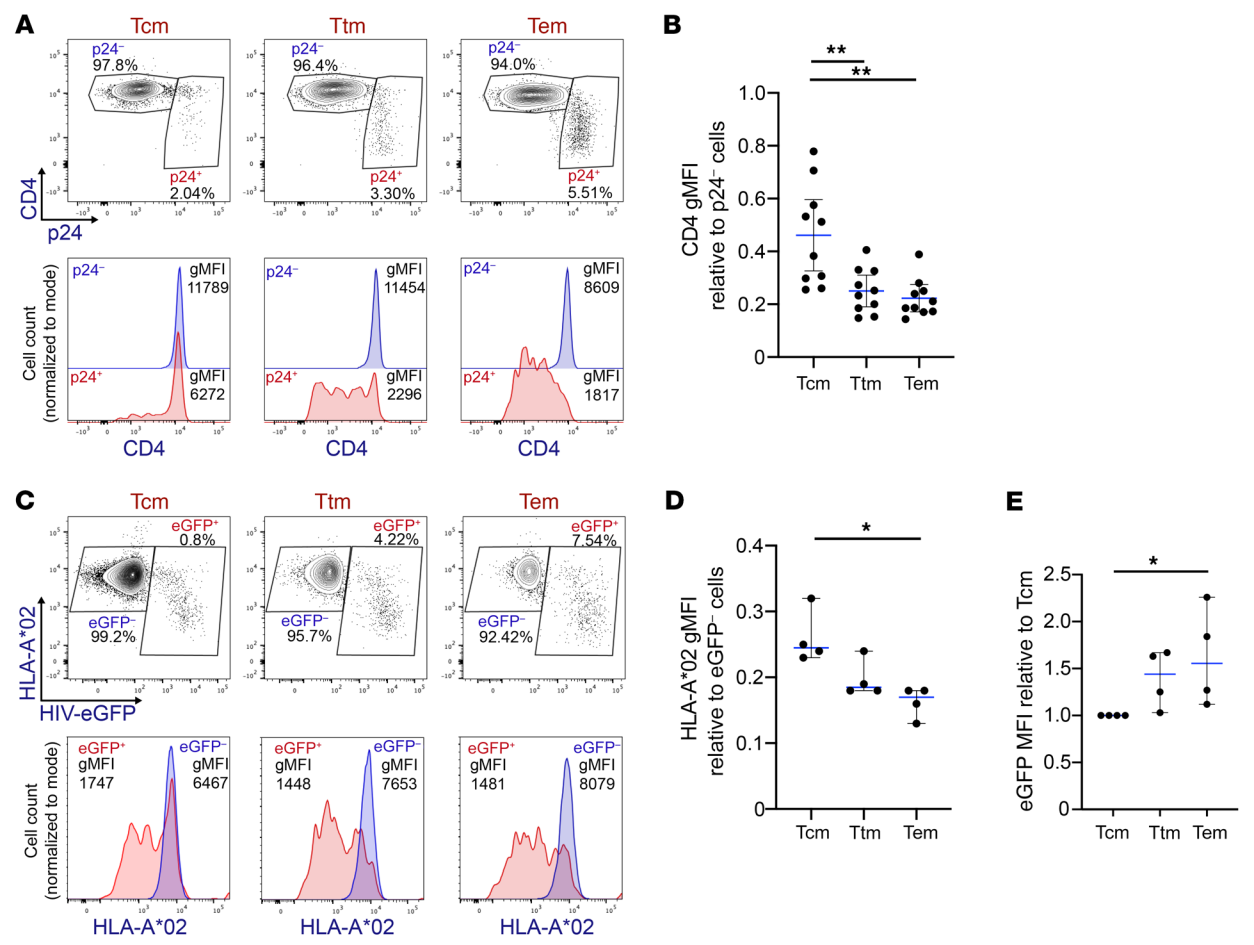
Nef activity is higher in HIV-1–infected CD4^+^ Tem cells. Memory CD4^+^ T cells from HIV-1^–^ donors were sorted and infected with (**A** and **B**) HIV-BaL or (**C**–**E**) HIV-NL4-3-eGFP for 5 days. (**A**) Representative dot plots (top) and histograms (bottom) of 10 independent experiments showing CD4 geometric mean fluorescence intensity (gMFI) in uninfected p24^–^ and HIV-1–infected p24^+^ cells. (**B**) CD4 expression in HIV-1–infected cells was calculated as CD4 gMFI in p24^+^ cells relative to CD4 gMFI in p24^–^ cells. (**C**) Representative dot plots (top) and histograms (bottom) of 4 independent experiments showing HLA-A*02 gMFI in uninfected eGFP^–^ and HIV-1–infected eGFP^+^ cells. (**D**) HLA-A*02 downmodulation in HIV-1–infected cells was calculated as HLA-A*02 gMFI in eGFP^+^ cells relative to HLA-A*02 gMFI in eGFP^–^ cells. (**E**) eGFP MFI relative to Tcm values. Data represent the mean ± SD (**B**) or median ± 95% CI (**D** and **E**). One-way ANOVA followed by the (**B**) Tukey’s HSD post test or (**D** and **E**) Kruskal-Wallis followed by Dunn’s post test were performed to determine statistical significance. Each data point represents a single donor. Tcm, central memory; Ttm, transitional memory; Tem, effector memory. *P ≤ 0.05, **P ≤ 0.01.

Our results indicate that Nef may protect cells harboring genetically intact *gag* and *pol* from CD8^+^ T cell clearance. Tcm cells containing genetically intact *gag* and *pol* correlated negatively with time on ART; however, this was not observed in Tem cells (Figure 3, C and D). We found that the proportion of sequences containing *gag*^+^*nef*^+^ in Tem cells was significantly higher than that in Tcm cells (*P* < 0.01; Figure 6D). Considering these findings, we hypothesized that Nef activity is higher in Tem cells compared with Tcm cells, making them less susceptible to CTL killing. To investigate this possibility, as a proxy for Nef activity, we quantified the downmodulation of CD4 from the cell surface of HIV-1–infected Tcm, Ttm, and Tem cells, because this molecule is predominantly downregulated by Nef (37). We sorted memory CD4^+^ T cell subsets from 10 HIV-1^–^ donors and infected them with HIV-1-BaL. At day 5 after infection, we performed CD4 and p24 immunostaining and quantified their expression by flow cytometry (Figure 10A). To determine whether the ability of HIV-1 to downregulate CD4 expression is different across the memory CD4^+^ T cell subsets, we quantified the geometric mean fluorescence intensity (gMFI) of CD4 in the population productively infected with HIV-1 (p24^+^) and compared it with the CD4 gMFI in the bystander, uninfected population (p24^–^) (Figure 10A). To quantify HIV-1–mediated CD4 downmodulation, we obtained the relative value of CD4 expression in the p24^+^ population (CD4 gMFI in p24^+^/CD4 gMFI in p24^–^) and compared this value among the Tcm, Ttm, and Tem cells (Figure 10B). Interestingly, we observed that the downmodulation of CD4 was higher in Tem cells, suggesting that the activity of Nef is higher in Tem cells when compared with Tcm cells.

Because Nef-mediated MHC-I downmodulation is associated with immune escape, we evaluated whether MHC-I downmodulation is different across the different CD4^+^ T cell subsets. Tcm, Ttm, and Tem cells were sorted from HIV-1^–^HLA-A*02^+^ donors and were infected with eGFP-expressing HIV-1 NL4-3 (Figure 10C). In agreement with the differential downregulation of CD4, we observed increased MHC-I downmodulation in Tem cells compared with Tcm cells (Figure 10D). As the eGFP gene was introduced into the *nef* ORF, we quantified the expression of eGFP/Nef in the different memory CD4^+^ T cell subsets (Figure 10E). Our results showed that Nef expression was higher in Tem cells. Moreover, the downmodulation of MHC-I molecules correlates with the relative level of eGFP expression (Supplemental Figure 5C). In addition, since it was reported that HIV-1 downmodulates the central memory markers CD27 and CCR7 (51, 52), we evaluated whether HIV-1–infected Tcm cells change their phenotype after viral infection in vitro. As shown in Supplemental Figure 6, Tcm cells retain their memory phenotype after HIV-1 infection. These results suggest that the increased downregulation of MHC-I observed in Tem cells can be associated with a higher expression of Nef within this cell subset.

Altogether, our results show that Tem cells are preferentially enriched for genetically intact HIV-1 proviruses after long-term ART, and we provide evidence that Nef contributes to the persistence of HIV-1 proviruses expressing immunogenic proteins such as Gag in this CD4^+^ T cell subset.

## Discussion

Identifying the cellular sources of the HIV-1 reservoir, predicting the dynamics of the HIV-1 proviral landscape, and determining the mechanisms of viral persistence are critical to finding new curative strategies and eradicating HIV-1 from infected individuals. To address these goals, our study presented 4 major results: (a) we showed that Tem cells are enriched for genetically intact HIV-1 proviruses in individuals on ART; (b) we found that the HIV-1 proviral landscape is different and may change over time within Tn and memory CD4^+^ T cell subsets; (c) we observed that CTL escape mutations do not contribute to HIV-1 persistence within Tem cells; and (d) we propose that HIV-1 Nef promotes persistence of genetically intact proviruses in this subset.

A growing body of evidence supports that memory CD4^+^ T cells are the main source of replication-competent HIV-1 (1, 9, 53–55). However, defining the contribution of specific CD4^+^ T cell subsets to the HIV-1 reservoir is ongoing. In this regard, we confirmed our previous observations that Tem cells are enriched for genetically intact and potentially replication-competent HIV-1 proviruses (4). In agreement with the findings of Venanzi Rullo et al., we also found that Tn cells contain a high proportion of genetically intact proviruses when compared with Tcm and Ttm cells (10). In contrast, Kwon and collaborators observed a similar distribution of genetically intact proviruses across resting Tn and memory CD4^+^ T cells from 10 HIV-1^+^ donors using the intact proviral DNA Assay (56). Differences observed between this and our study may be due to the number of participants and sequences analyzed.

In addition, we characterized the HIV-1 proviral landscape based on the proportion of different classes of full-length and deleted sequences. Of note, this is the first study to our knowledge to utilize in-depth near-full-length sequencing and vigorous statistical analysis to compare the proviral landscape between cell subsets in a large cohort of participants. Our results showed that the proviral landscape is different across Tn and memory CD4^+^ T cells. Because we and others have shown that cells infected with defective proviruses can produce viral proteins and be targeted by CTLs (17, 57), characterizing the HIV-1 proviral landscape can help our understanding of how intact and defective proviruses persist in the different CD4^+^ T cell subsets. For instance, the proportion of HIV-1 proviral sequences with predominantly 5′ deletions is higher in Tem cells. Interestingly, the 3′ genomic region contains the viral gene *nef*, which is a key viral factor that may contribute to the persistence of these proviruses in this particular CD4^+^ T cell subset.

Although viral evolution and viral sequence diversity remain stable after long-term effective ART (58–61), when we examined the relationship between different types of defective proviruses and ART duration in a interparticipant cross-sectional analysis, we found that the HIV-1 proviral landscape may change over time, and these changes are different between Tn and memory CD4^+^ T cell subsets. The dynamics of the proviral landscape have been studied previously, showing that genetically intact proviruses and sequences containing genes coding immunogenic proteins, such as Gag, decrease over time (10, 18–21, 47). However, most of these studies did not examine the dynamics of the proviral landscape across CD4^+^ T cell subsets. In agreement with previous publications, our findings suggest that the proportion of genetically intact HIV-1 proviruses in Tn CD4^+^ T cells declined over time (10). Interestingly, we showed that in Tcm cells the proportion of sequences with genetically intact *gag* and *pol* ORFs correlated negatively with ART duration. Although others have shown that proviruses with intact *gag* and *gag-pol* regions decrease over time on ART in total CD4^+^ T cells (18, 20), this is the first time to our knowledge that this has been observed exclusively in Tcm cells; this may be explained by the significant contribution Tcm cells make to the total CD4^+^ T cell population.

Interestingly, we found that in Tcm cells the proportion of Gag and Pol sequences harboring WT CTL epitopes correlated negatively with ART duration. Additionally, the proportion of sequences with unrecognizable epitopes correlated positively with ART duration, suggesting that the potential decline in sequences containing genetically intact *gag* and *pol* ORFs in Tcm cells may be driven by CD8^+^ T cell activity. However, when the proportion of sequences harboring Nef-derived WT epitopes was analyzed, we found a low proportion of these epitopes and no correlation was observed with time on ART. This may be a consequence of Nef immunodominance in the acute phase of HIV-1 infection (62–64), where proviruses harboring Nef-derived WT epitopes can be quickly targeted by CTLs. Surprisingly, it was reported that Tcm cells are more resistant to the activity of CTLs when compared with Tem cells (22, 24, 65), indicating that immune pressure is likely stronger in Tem cells. However, the target cells in these studies were generated by pulsing cells with peptides rather than infecting them; therefore, the contribution of viral factors, such as immunomodulatory viral proteins, in protecting the cells from CTL activity was not considered.

As CTL escape mutations and unrecognizable epitopes were not enriched in the Tem subset, we hypothesized that the persistence of genetically intact proviruses and sequences containing intact *gag* and *pol* ORFs in Tem cells must be mediated by a separate mechanism. In addition to CTL escape mutations, HIV-1 expresses proteins with immunomodulatory functions, such as Nef. The role of Nef in immunopathogenesis and immune evasion has been extensively reported (33, 66–72). To determine whether Nef plays a role in promoting the persistence of infected Tem cells, we analyzed the proportion of sequences with an intact *nef* ORF in Tn and memory CD4^+^ T cell subsets. Of note, we observed a higher proportion of sequences with an intact *nef* ORF in Tem cells. We also observed that the proportion of sequences containing genetically intact *gag* or *pol* in combination with *nef* is significantly higher in this subset when compared with Tcm cells. Although most of the proviruses found in HIV-1–infected donors on ART are defective, in agreement with Imamichi et al. (57), we provide evidence that HIV-1 proviruses containing large internal deletions can express both Nef and Gag. This indicates that Nef expression from defective proviruses may lead to MHC-I downmodulation and protect cells expressing viral proteins from CTLs. Moreover, our ex vivo analysis revealed that the proportion of proviruses containing *gag* or *pol* in combination with *nef* increased after CD8^+^ T cell clearance. Our in vivo and ex vivo results suggest that Nef activity affects the proviral HIV-1 landscape, at least, in Tem cells. By quantifying the HIV-1–mediated downmodulation of CD4 and MHC-I as a proxy for Nef activity, we found that the activity of this viral protein is higher in Tem cells compared with Tcm cells. In addition, these differences were associated with higher expression of Nef in Tem cells, supporting the role of Nef in this cell subset. In agreement with findings of earlier studies, our findings suggest that targeting Nef can be an attractive strategy for reducing HIV-1 persistence during therapy by improving CTL responses. Recent promising studies have investigated the effects of Nef blockade on reducing HIV-1 infection and enhancing CD8^+^ T cell clearance (71, 73–78).

One limitation of our study is that due to the complexity of HIV-1 protein expression, in the absence of direct measurement, whether each individual provirus harboring an intact ORF can express a functional protein remains unknown. However, we showed Gag and Nef expression from 7 viral constructs, based on participant sequences that contained large internal deletions, which supports work by others who have demonstrated protein expression from defective proviruses (17, 57, 79). Another limitation of our study is that the expression of Nef was not quantified in the CD4^+^ T cells from which our proviral sequences were derived. However, recent observations in longitudinal assessments have suggested that Nef expression persists during long-term ART (72, 80). Furthermore, recent studies have demonstrated that the size and dynamics of the HIV-1 reservoir may be different between males and females. As only one female was included as a participant in this study, we were unable to investigate how biological sex influences the distribution, diversity, and persistence of HIV-1 in individuals on long-term ART, and this deserves further study. A final limitation of our study is that we performed an interparticipant cross-sectional analysis, rather than a longitudinal study, due the limited availability of samples with multiple time points. Nevertheless, the analysis of samples from 24 participants over 17 years of ART allowed us to investigate the relationship between the HIV-1 proviral landscape and time of ART. Moreover, our experimental data support the hypotheses derived from our sequence analysis.

In conclusion, our study shows that the HIV-1 proviral landscape is different across Tn and memory CD4^+^ T cell subsets. Importantly, we show that Tem cells are a key component of the HIV-1 reservoir after long-term ART, since this subset is enriched for genetically intact HIV-1 proviruses. Moreover, our findings reveal that *nef* contributes to the persistence of genetically intact and defective HIV-1 proviruses, particularly in Tem cells during ART. This suggests that targeting Nef would enhance CTL-mediated immune clearance of HIV-1–infected cells during effective therapy.

## Methods

### Participant cohort and clinical samples.

Twenty-four participants from SCOPE and OPTIONS long-term observational cohorts, based at the University of California, San Francisco, were included in this study. All participants were HIV-1 subtype B^+^, on suppressive ART (1.8–17.7 years), and had maintained viral suppression (viral load <75 HIV-1 RNA copies per ml) for at least 1 year prior to the donation of samples (Supplemental Table 1).

### Isolation of CD4^+^ T cell subsets.

All participants provided blood or leukapheresis samples from which PBMCs were isolated by Ficoll-Hypaque density gradient centrifugation. CD4^+^ T cells were isolated from PBMCs by negative selection and FACS to obtain Tn, Tcm, Ttm, and Tem CD4^+^ T cell subsets. The sorting strategies used to isolate Tn and memory CD4^+^ T cells have been described previously (4, 28, 60). The specific markers used to obtain Tn and memory CD4^+^ T cell subsets for each participant are outlined in Supplemental Table 7.

### FLIPS.

Near-full-length HIV-1 proviral sequences were obtained using the FLIPS assay as previously described (4, 12).

### Identification of intact, defective, and identical proviruses.

Proviruses were identified as intact using the Proviral Sequence Annotation & Intactness Test (https://psd.cancer.gov/tools/tool_index.php) with the following additional criteria: (a) required all ORFs to have an intact start codon (18); (b) required intact ORFs for regulatory/accessory proteins (absence of deletions/insertions >5% length of ORF, stop codons, or frame shift mutations); (c) allowed for the presence of a cryptic MSD (4 nucleotides downstream of the MSD 1) to salvage a mutated MSD 1 (81).

Defective HIV-1 proviruses were characterized as full-length (>8800 bp), deleted (<8800 bp), or containing a genomic rearrangement (such as an inversion or repeated region). Full-length proviruses were then sequentially characterized as hypermutants using the Los Alamos Hypermutation tool (https://www.hiv.lanl.gov/content/sequence/HYPERMUT/hypermut.html); containing a *cis*-acting defect (a mutation in the MSD or deletion within any of the 4 stem loops of the packaging signal [HXB2 position 695–810]); containing a frame shift mutation in any ORF; or containing a single stop codon in any ORF. Deleted proviruses were characterized as containing a predominantly 5′ deletion; a predominantly 3′ deletion; a central deletion; or a deletion >75% of the length of HXB2 (proviruses <2430 bp in length). Note that due to the low proportion of proviruses with frame shift mutations, single stop codons, and central deletions, these categories of proviruses were not considered for further analysis.

ORFs were identified as intact if they contained an intact start codon in the absence of deletions/insertions >5% length of ORF, stop codons, or frame shift mutations. Sequences containing a genomic rearrangement were not included in the ORF analyses. Splice donor and acceptor sites were not considered in the identification sequences with intact ORFs due to (a) the genetic diversity of splice sites across the participants; (b) previous studies showing HIV-1 protein expression from proviruses with defective splice sites (17, 57, 79); and (c) evidence that HIV-1 can utilize cryptic splice sites when traditional splice sites are defective (17).

Sequences were identified as identical if (a) they were identified as 100% identical by the Los Alamos ElimDupes tool (https://www.hiv.lanl.gov/content/sequence/elimdupesv2/elimdupes.html) or (b) they were of the same length with identical deletion junction sites.

### Epitope and CTL escape mutation analysis.

To quantify the proportion of CTL epitopes from Gag, Pol, and Nef for each participant, the Los Alamos “Best-defined CTL/CD8^+^ Epitope Summary” was used (https://www.hiv.lanl.gov/content/immunology/tables/optimal_ctl_summary.html). This is a regularly updated list of experimentally tested HLA class I–restricted epitopes. For each viral sequence, we identified WT epitopes, escape variants, and unrecognizable CTL epitopes. Unrecognizable epitopes were classified as those lacking a start codon and/or containing premature stop codons and/or frame shift mutations. Escape variants were identified using the Los Alamos “CTL/CD8^+^ Epitope Variants and Escape Mutations” table (http://www.hiv.lanl.gov/content/immunology/variants/ctl_variant.html), which summarizes published data on epitope variants and their restricted HLA type. Only epitopes and escape variants validated experimentally were considered in our analysis.

To identify WT epitopes, escape variants, and unrecognizable epitopes, the Biopython SeqUtils package (https://github.com/biopython/biopython; commit id: e001f2eed6a9cc6260f60f87ed90fcf8ea2df4ca) was used, and data were analyzed in R (v. 4.0.2).

### Peptide pools.

Potential T cell epitope peptide panels corresponding to Pol (catalog 12961) and Gag (catalog 12437) HIV-1 proteins were obtained from the NIH HIV Reagent Program, Division of AIDS, NIAID, NIH, Bethesda, Maryland, USA.

### Plasmids and HIV-1 viral strains.

The molecular clones HIV-1 NL4-3^AD8ENV^ (HIV-WT), HIV-1 BaL, and pHEF-VSVg were obtained through the NIH AIDS Reagent Program. HIV^NEF-ve^ (HIV-ΔNef) and HIV-NL4-3-eGFP clones were provided by Stuart G. Turville (82).

HIV-1 NL4-3 constructs containing large internal deletions (NL-1408_09, NL-2208_02, NL-2278_24, NL-2452_22, NL-2531_09, NL-2531_11, NL-2531_19) were generated by using gBlocks Gene Fragments (Integrated DNA Technologies). Briefly, gBlocks containing deletions were amplified by PCR, the amplicons were digested using specific restriction enzymes (New England Biolabs) and ligated to the previously digested NL4-3 backbone using T4 DNA ligase (Promega). Finally, the resulting plasmids were amplified transforming Stbl2 bacteria (Thermo Fisher Scientific). HEK-293T cells (ATCC, CRL-11268) were transfected with these constructs to perform flow cytometry or immunoblotting.

### Immunoblotting.

Cells were lysed in RIPA buffer (Abcam) supplemented with a cocktail of antiproteases (Roche), and nuclei were cleared by centrifugation at 15,000*g* for 10 minutes at 4°C. Ten μg of protein extracts were separated by 4%–15% SDS-PAGE (Mini PROTEAN TGX Stain-Free Gels, Bio-Rad) and blotted on PVDF Transfer Membrane (Trans-Blot Turbo transfer pack, Bio-Rad) under reducing conditions. After membrane blocking (Intercept PBS Blocking Buffer, LI-COR), membranes were incubated with mouse anti–β-Actin (sc-8432, Santa Cruz Biotechnology) or mouse anti-p24 (clone KC-57, Beckman Coulter) and rabbit anti-Nef (catalog 2949, obtained through the NIH AIDS Reagent Program) for 90 minutes. Then, membranes were washed and incubated with goat anti-mouse 700 (catalog no. 926-68070, LI-COR) and goat anti-rabbit 800 (catalog no. 926-32211, LI-COR) for 1 hour. Blots were revealed using an Odyssey DLx Imaging System reader (LI-COR).

### Preparation of virus stocks.

HIV-WT, HIV-ΔNef, and HIV-NL4-3-eGFP viral stocks were produced by transfection of the corresponding vector (1 μg/well) in HEK293T cells (3 × 10^5^ cells per well in 6-well plates) using X-treme gene transfection reagent (Roche). Pseudotyping was achieved by cotransfecting pHEF-VSVg (400 ng/well). Supernatant was harvested at 48 and 72 hours after transfection, cleared by centrifugation at 1500g for 10 minutes, and frozen at –80°C. The titer of the virus stock was measured by flow cytometry analysis of p24 expression 72 hours after infection of Jurkat cells (obtained through the NIH HIV Reagent Program).

Purified high-titer stocks of the CCR5-tropic HIV-BaL strain were produced as described previously (83).

### HIV-1 infection in vitro of isolated CD4^+^ T cells.

Isolated CD4^+^ T cells (obtained as described below) were cultured at a concentration of 10^6^ cells/ml in cRF10 (RPMI, Lonza) and 10% fetal bovine serum (RF10), supplemented with 1× GlutaMAX (Gibco), 100 U/ml penicillin (Gibco), and 100 μg/ml streptomycin (Gibco) in a 96-well U-bottom plate and incubated with the corresponding HIV-1 stock overnight at 37°C as previously described (84). Then, cells were washed 3 times with PBS, and cRF10 was added. Cells infected with HIV-1-NL4-3-eGFP were spinoculated by centrifugation at 800*g* for 90 minutes in the presence of 8 μg/ml Polybrene (MilliporeSigma).

### Isolation and cell sorting of memory CD4^+^ T cell subsets for infection in vitro.

Isolation of memory CD4^+^ T cells to be infected with HIV-BaL and HIV-NL4-3-eGFP was performed as previously described (84).

### HIV-1–specific CD8^+^ T cell expansion.

The expansion of HIV-1–specific CD8^+^ T cells was adapted from the Salido et al. model (85, 86). Cryopreserved PBMCs were thawed, rested overnight, and then cultured in 24-well plates at a density of 2 × 10^6^ cells/ml in cRF10 in the presence of 4 μg/ml of the corresponding HIV-1 peptide pool for 1 to 2 hours. Next, cells were washed with RF10 and cultured with cRF10 overnight, followed by culture with cRF10 supplemented with 100 U/ml IL-2 (Biolegend Inc.), 200 U/mL IL-7 (Miltenyi Biotec), and 200 U/ml IL-15 (Miltenyi Biotec) for 12 days. Media was replaced every 48–72 hours. Expanded cells were purified by negative selection (Human CD8^+^ T cell Isolation Kit, Miltenyi Biotec) and subsequently cocultured with autologous HIV-1–infected CD4^+^ T cells.

### Generation of autologous CD4^+^ T cell targets.

PBMCs were thawed and cultured in cRF10 medium supplemented with 100 U/ml IL-2, 0.5 μg CD3/8 bispecific antibody (ARP-12277, obtained from Johnson Wong and Galit Alter through the NIH AIDS Reagent Program), and antiretrovirals (100 nM Efavirenz, 100 ng/ml Enfuvirtide and 30 mM Raltegravir, MilliporeSigma) to avoid HIV-1 spread and reinfection. PBMC treatment with CD3/8 bispecific antibody resulted in the elimination of CD8^+^ T cells and enrichment of activated CD4^+^ T cells. Purification of CD4^+^ T cells was performed by negative selection using magnetic beads (Human CD4^+^ T cell Isolation Kit, Miltenyi Biotec).

### Viral competition assay.

Isolated CD4^+^ T cells obtained from HIV-1^+^ donors (as detailed above) were infected with HIV-WT or HIV-ΔNef. Cells were stained with the dye CellTrace Far Red (Thermo Fisher Scientific) at 100 nM (HIV-ΔNef) and 1 μM (HIV-WT) to identify each population of infected cells by flow cytometry. After 24 hours of infection, antiretrovirals were added to avoid HIV-1 spread and reinfection (100 nM Efavirenz, 100 ng/ml Enfuvirtide, MilliporeSigma). On day 3 after infection, infected cells were mixed and cocultured with autologous expanded CD8^+^ T cells at a 1:3 to 1:20 CD4/CD8 ratio. After 24 hours, one-half of the culture was used to measure p24 expression by flow cytometry. In parallel, the second half was washed with PBS and then incubated with 60 U DNase I for 30 minutes at 37°C to degrade the HIV-1 proviral DNA from dead cells. Next, cells were washed 3 times with PBS before performing cell lysis and FLIPS.

### Data availability.

The GenBank accession numbers for sequences reported in this study are as follows: KY778264–KY778681, KY766150–KY766212, MW754554–MW754712, MZ080627–MZ081008, MN466964–MN467397, MZ922480–MZ923010, and OL872744–OL873105. In-house Python scripts are available from the authors upon request.

### Statistics.

The estimated proportion of sequenced intact HIV-1 proviruses by cell subset was calculated using a mixed logistic model to adjust for the repeated observations within each participant. A random effect for all terms (both intercept and subset) was included. *P* values for the fixed effect of subset were calculated with a likelihood ratio test. The comparison of proportions of defective proviruses and ORFs between subsets was performed using a mixed logistic model. Pairwise comparisons were conducted between cell subsets, and a likelihood ratio test was used to assess the significance of the difference between subsets. Subsets with <5 total sequences were excluded from analysis. As most of the performed comparisons are not independent, the reported *P* values were not adjusted for multiple comparisons, since this correction assumes independence of the comparison, and this would inflate type 2 errors. Mixed logistic regression was performed in R: a language and environment for statistical computing, version 4.0.4 (library lme4, function glmer).

We used the nonparametric Spearman’s correlation test in Prism (GraphPad Software) for correlation analyses. Where data were normally distributed (according to the Kolmogorov-Smirnov test), we applied single linear regression.

Data from in vitro and ex vivo experiments were analyzed using Prism (GraphPad Software). Normality of the data was tested using the Kolmogorov-Smirnov test. Based on the normality test, either 1-way ANOVA followed by the Tukey’s HSD post test or Kruskal-Wallis followed by Dunn’s post test were used for multiple comparison analyses, and a 2-tailed Student’s *t* test was used to compare 2 conditions. A *P* value less than 0.05 was considered significant.

### Study approval.

This study was approved by the institutional review boards at the University of California, San Francisco, and the Western Sydney Local Health District, which includes the Westmead Institute for Medical Research. Written informed consent was obtained from all participants.

## Author contributions

GD, BH, and SP designed the study. BH conducted the proviral landscape analyses. GD designed and conducted ex vivo and in vitro experiments. HM and TES conducted and interpreted the statistical analysis. VM and ST provided HIV-1 constructs. FGM and GD designed and conducted CTL epitope analysis. BH, BAH, and KF conducted FLIPS on participant samples. OT, NN, and ALC provided experimental data. EL, HA, and AS prepared participant samples. RH, SGD, NC, RF, CBS, PWH, and ADK enrolled the participants and collected and/or sorted cell subsets from the participant samples. GD and BH wrote the original manuscript. SP supervised the work and edited the manuscript. Co–first authorship order was determined by the contribution to the results presented in the manuscript and contribution to writing the initial manuscript draft.

## Supplementary Material

Supplemental data

## Version 1 02/08/2022

In-Press Preview

## Version 2 04/01/2022

Electronic publication
