# Supplementary material for: The HIV-1 proviral landscape reveals that Nef contributes to HIV-1 persistence in effector memory CD4+ T cells
Source: J Clin Invest. 2022 Apr 1;132(7):e154422. doi: 10.1172/JCI154422 (PMC8970682; doi:10.1172/JCI154422)
Supplement: Supplemental data [file jci-132-154422-s180.pdf]

Supplemental Material

The HIV-1 proviral landscape reveals Nef contributes to HIV-1 persistence in effector memory CD4+ T-cells

Gabriel Duette, Bonnie Hiener, Hannah Morgan, Fernando G. Mazur, Vennila Mathivanan, Bethany A. Horsburgh, Katie Fisher, Orion Tong, Eunok Lee, Haelee Ahn, Ansari Shaik, Rémi Fromentin, Rebecca Hoh, Charline Bacchus-Souffan, Najla Nasr, Anthony Cunningham, Peter W. Hunt, Nicolas Chomont, Stuart Turville, Steven G. Deeks, Anthony D. Kelleher, Timothy E. Schlub, and Sarah Palmer.

Supplementary Table 1. Participant characteristics

Participant ID	Age	Sex	ART duration[A] (years)	Time of infection before initiation of therapy (months)	CD4+ T-cell count[A] (cells per µl)	Viral load[A] (copies/ml)	Therapeutic regimen
3632	31	Male	1.8	20.8	902	<40	EVG/COBI/FTC/TDF
2664	46	Male	2.7	4.1	637	<40	EVG/COBI/FTC/TDF
1408	31	Male	3.1	45.5	637	<40	EFV/FTC/TDF
2452	66	Male	3.2	24.4	604	<40	MVC, RTG, ETR
2647	33	Male	3.4	4.5	532	<40	FTC/TDF, RTV, DRV
2531	51	Male	3.4	1.9	1163	<40	FTC/TDF, RGV
2606	29	Male	3.5	1.7	787	<40	ABC/TCV/3TC
1292	44	Male	3.5	81.8	746	<40	EFV/FTC/TDF
1756	29	Male	4.1	6.8	582	<40	RPV/TDF/FTC, TCV
2470	45	Male	4.5	15.6	602	<40	ATV, RTV, TDF/FTC
2302	27	Male	4.6	3.4	696	<40	FPV, RTV, TDF/FTC
2303	39	Male	4.8	2.8	606	<40	EFV/FTC/TDF
2469	41	Male	5.7	30.5	1089	<40	ABC/3TC, ATV
2241	56	Male	6.4	30.7	569	<40	EFV/FTC/TDF
2454	35	Male	7.1	0.7	513	<40	RPV/TDF/FTC
2208	64	Male	7.1	114.5	437	<40	FTC/TDF, ATV, RTV
2278	43	Male	7.8	4.7	883	<40	FTC/TDF, NVP
2286	46	Male	9.1	1.3	381	<40	EFV/FTC/TDF
2274	54	Male	11.8	13.1	486	<40	FTC/TDF, NVP
2275	47	Male	15.3	0.8	1842	<40	FTC/TDF, NVP
2518	55	Female	15.3	118.3	432	<40	TDF, AZT/3TC, NVP
2046	50	Male	16.3	69.9	1099	<40	ECV,EFV/TDF/FTC
2115	51	Male	17.3	0.6	601	<40	FTC/TDF, NVP
2026	59	Male	17.7	117.0	476	<40	TDF, ABC/3TC, RTV, DRV

3TC, lamivudine; ABC, abacavir; ATV, atazanazir; AZT, zidovudine; COBI, cobicistat; DRV, darunavir; ECV, entecavir; EFV, efavirenz; ETR, Etravirine; EVG, elvitegravir; FPV, fosamprenavir; FTC, emtricitabine; MVC, maraviroc; NVP, nevirapine; RPV, rilpivirine; RTG, raltegravir; RTV, ritonavir; TCV, dolutegravir; TDF, tenofovir disoproxil fumarate

[A]At time of sampling.

Supplementary Table 2. Number of cells and sequences obtained for each subset and participant

Participant ID	T_N			T_{CM}			T_{TM}			T_{EM}		
	Defective	Intact	No. of Cells Analyzed	Defective	Intact	No. of Cells Analyzed	Defective	Intact	No. of Cells Analyzed	Defective	Intact	No. of Cells Analyzed
2302	15	4	3287933	34	0	704122	30	3	631348	32	6	728025
2115	-	-	-	28	0	2294141	29	0	859259	20	3	1888889
2275	-	-	-	3	0	251852	20	0	1057688	34	1	1330640
2046	0	0	6465	24	0	190647	1	0	4048	23	2	153236
2452	35	2	641975	32	0	908333	37	0	225734	33	2	459055
2026	26	0	152727	1	0	47685	10	1	133448	32	1	51046
2469	1	0	1824159	33	0	1459476	37	0	960696	34	1	500022
2278	9	0	17385254	45	0	4564677	30	0	3295980	35	1	1606036
2286	13	0	906846	50	0	1997611	42	0	740607	26	0	929653
2303	44	6	7433615	27	1	2994562	48	5	1191647	40	10	1611478
1292	32	2	1697256	63	2	434237	50	0	418886	65	0	979955
2470	28	2	2035499	23	0	1638093	23	0	1286067	24	1	1037773
2241	16	4	1896296	24	0	1178519	25	1	955485	24	0	1325583
2647	20	1	2008627	49	2	606738	35	0	488060	46	2	557995
2531	2	1	846802	26	0	845044	34	0	369349	37	0	241380
2664	12	0	2460392	42	0	1124804	24	1	750758	56	0	285373
2606	-	-	2377831	10	0	3074632	34	1	2709673	21	0	1419446
2454	7	0	944567	34	0	822853	42	1	606953	26	0	370087
1408	1	0	2008675	39	0	666109	46	0	288519	20	6	196068
3632	6	2	1972938	14	0	844189	31	2	1200376	35	0	409945
1756	-	-	2860172	15	0	1024396	19	15	997705	18	22	679114
2274	-	-	342384	25	1	1107273	43	0	825005	25	10	350637
2208	36	0	1010249	35	1	367777	31	2	419586	34	0	267978
2518	35	0	1040000	38	0	77217	48	0	39506	38	0	941235
**Total**	**338**	**24**	**55140662**	**714**	**7**	**29224986**	**769**	**32**	**20456383**	**778**	**68**	**18320648**

Supplementary Table 3. Number of sequences obtained for each category of HIV-1 provirus

	Cell subset	Total*	Total (ex. inversions)	Intact	Full-length	Hypermut-ant	*cis*-acting defect	5' deletion	3' deletion	>75% deleted	*gag*+	pol+	nef+	*gag*+nef+	pol+nef+
**2302**	T_N	19	17	4	5	0	0	2	3	7	10	7	7	4	4
	T_{CM}	33	31	0	7	7	0	2	7	13	4	1	3	0	0
	T_{TM}	32	31	3	6	3	0	3	8	13	8	4	5	3	3
	T_{EM}	30	30	6	8	1	1	3	6	13	12	8	10	7	7
**2115**	T_N	0	0												
	T_{CM}	25	23	0	13	13	0	2	5	3	2	0	3	1	0
	T_{TM}	13	12	0	5	5	0	1	3	3	3	0	0	0	0
	T_{EM}	16	15	2	9	7	0	2	2	2	4	2	3	2	2
**2275**	T_N	0	0												
	T_{CM}	3	1												
	T_{TM}	12	12	0	4	3	1	3	2	3	2	1	6	1	1
	T_{EM}	11	10	1	3	0	1	1	1	5	3	3	3	2	2
**2046**	T_N	0	0												
	T_{CM}	20	13	0	1	1	0	1	4	7	5	0	0	0	0
	T_{TM}	1	0												
	T_{EM}	6	6	1	2	0	1	1	2	1	4	2	3	2	2
**2452**	T_N	37	32	2	7	5	0	0	6	17	6	3	4	2	2
	T_{CM}	30	29	0	2	2	0	1	12	14	12	2	0	0	0
	T_{TM}	8	7	0	1	0	1	0	1	5	2	1	3	1	1
	T_{EM}	14	14	2	3	0	0	1	4	5	8	4	4	3	2
**2026**	T_N	22	21	0	1	0	1	0	7	13	7	1	4	1	0
	T_{CM}	1	1												
	T_{TM}	9	9	1	2	0	1	0	3	4	3	2	3	2	2
	T_{EM}	6	6	1	2	0	1	1	1	2	3	2	4	2	2
**2469**	T_N	1	1												
	T_{CM}	32	29	0	0	0	0	2	7	19	5	1	4	2	1
	T_{TM}	31	28	0	2	2	0	4	7	14	6	0	8	2	0
	T_{EM}	13	11	1	2	1	0	1	2	6	3	1	4	1	1
**2278**	T_N	9	8	0	2	1	1	1	1	4	2	1	2	0	0
	T_{CM}	43	40	0	2	1	1	4	8	26	11	2	4	2	1
	T_{TM}	28	25	0	2	2	0	3	6	14	7	0	3	0	0
	T_{EM}	23	20	1	3	2	0	2	8	7	5	1	5	2	1
**2286**	T_N	13	13	0	3	3	0	0	6	4	7	1	2	0	0
	T_{CM}	41	40	0	1	0	1	1	5	33	4	1	7	1	1
	T_{TM}	35	34	0	0	0	0	5	4	25	4	0	6	0	0
	T_{EM}	12	9	0	2	0	2	1	2	4	4	2	2	2	2
**2303**	T_N	46	44	6	16	10	0	2	17	9	22	9	13	8	6
	T_{CM}	28	28	1	7	6	0	0	11	10	11	3	5	1	1
	T_{TM}	22	18	3	6	2	0	2	2	8	8	3	5	4	3
	T_{EM}	18	16	6	7	0	1	2	2	5	7	7	9	7	7
**1292**	T_N	34	31	2	2	0	0	0	10	19	14	2	7	3	2
	T_{CM}	61	56	2	6	3	1	0	17	33	19	2	8	5	2
	T_{TM}	32	30	0	0	0	0	0	10	20	7	1	4	2	0
	T_{EM}	43	40	0	0	0	0	3	13	24	9	1	9	0	0
**2470**	T_N	28	28	2	5	3	0	1	11	11	14	3	7	4	2
	T_{CM}	22	21	0	0	0	0	2	9	10	8	0	2	0	0
	T_{TM}	21	20	0	0	0	0	1	2	17	3	1	1	0	0
	T_{EM}	8	7	1	1	0	0	1	1	4	2	1	2	1	1

**2241**	T_N	20	19	4	5	0	1	1	7	6	12	7	7	4	4
	T_{CM}	23	20	0	1	1	0	1	5	13	5	1	4	0	0
	T_{TM}	21	19	1	3	2	0	0	6	10	9	2	3	1	1
	T_{EM}	19	17	0	0	0	0	0	6	11	4	0	3	0	0
**2647**	T_N	19	19	1	4	3	0	1	5	9	6	1	6	2	1
	T_{CM}	47	43	2	2	0	0	2	16	23	11	4	13	4	2
	T_{TM}	33	29	0	2	1	1	0	8	18	10	1	6	2	1
	T_{EM}	38	36	2	5	3	0	3	8	20	9	2	8	2	2
**2531**	T_N	3	3												
	T_{CM}	26	26	0	3	1	2	0	15	8	19	4	6	4	2
	T_{TM}	34	30	0	5	3	1	0	15	10	17	5	3	3	1
	T_{EM}	30	29	0	3	2	1	4	17	5	15	0	8	3	0
**2664**	T_N	11	11	0	0	0	0	0	1	10	1	1	1	0	1
	T_{CM}	41	40	0	4	4	0	3	11	22	14	3	4	0	0
	T_{TM}	23	23	1	5	4	0	0	6	12	8	2	1	1	1
	T_{EM}	22	21	0	0	0	0	2	5	14	3	0	5	0	0
**2606**	T_N														
	T_{CM}	10	7	0	0	0	0	1	1	5	3	0	2	0	0
	T_{TM}	29	29	1	2	0	0	2	12	12	17	2	5	4	1
	T_{EM}	17	16	0	2	2	0	0	8	6	5	0	1	0	0
**2454**	T_N	7	7	0	2	2	0	1	3	1	4	0	3	2	0
	T_{CM}	34	34	0	7	7	0	0	15	11	11	2	10	5	1
	T_{TM}	41	40	1	7	5	1	0	14	17	14	3	7	6	2
	T_{EM}	18	18	0	4	3	1	4	4	5	5	2	7	1	1
**1408**	T_N	1	1												
	T_{CM}	38	37	0	2	2	0	5	16	14	16	1	8	3	0
	T_{TM}	37	36	0	1	0	1	1	15	18	15	1	14	5	0
	T_{EM}	19	19	3	4	0	1	3	4	4	6	4	13	5	4
**3632**	T_N	8	8	2	3	1	0	1	0	3	2	2	3	2	2
	T_{CM}	14	11	0	4	0	1	1	4	2	6	2	8	4	2
	T_{TM}	29	26	2	6	2	2	2	5	13	8	2	7	2	2
	T_{EM}	17	16	0	6	0	6	4	0	6	3	3	9	3	3
**1756**	T_N														
	T_{CM}	14	13	0	3	2	1	0	6	4	7	2	1	1	1
	T_{TM}	23	23	5	7	0	2	1	7	8	13	8	11	7	7
	T_{EM}	16	16	11	14	0	1	1	0	1	14	12	15	14	12
**2274**	T_N	0	0												
	T_{CM}	25	20	1	8	6	0	0	6	5	6	1	4	2	1
	T_{TM}	43	39	0	4	4	0	6	16	12	15	1	9	1	0
	T_{EM}	13	12	1	2	0	1	1	4	5	5	2	5	2	2
**2208**	T_N	36	34	0	9	7	1	1	10	14	11	1	4	2	1
	T_{CM}	27	27	1	5	3	1	2	11	9	8	3	6	1	1
	T_{TM}	27	26	2	3	0	1	3	9	11	7	2	9	2	2
	T_{EM}	12	11	0	2	0	2	1	3	5	5	2	4	3	2
**2518**	T_N	34	30	0	0	0	0	0	14	16	15	0	7	4	0
	T_{CM}	35	34	0	1	0	1	1	25	7	24	1	7	2	0
	T_{TM}	11	9	0	0	0	0	0	5	4	5	0	2	0	0
	T_{EM}	3	2	0	0	0	0	1	0	1	1	0	1	0	0

*Total unique sequences (identical sequences counted once for each cell subset)

Subsets with < 5 total sequences excluded from analysis

Supplementary Table 4. Spearman correlation analyses for relationship between types of defective proviruses and time on ART

Proviral category	Subset	r	p-value
Full-length	T_N	-0.11	0.70
	T_{CM}	0.02	0.92
	T_{TM}	0.15	0.50
	T_{EM}	0.38	0.08
Hypermutant	T_N	0.05	0.87
	T_{CM}	0.22	0.34
	T_{TM}	0.16	0.47
	T_{EM}	0.08	0.73
cis-acting defect	T_N	0.50	0.06
	T_{CM}	0.00	0.99
	T_{TM}	-0.15	0.52
	T_{EM}	0.33	0.14
5' Deletion	T_N	-0.14	0.60
	T_{CM}	-0.15	0.51
	T_{TM}	0.37	0.09
	T_{EM}	0.09	0.69
Deletion > 75%	T_N	-0.17	0.55
	T_{CM}	-0.16	0.48
	T_{TM}	-0.45	**0.03**
	T_{EM}	-0.30	0.18

Supplementary Table 5. Deletion positions for participant sequences reproduced in NL4-3 proviral construct

Participant sequence	NL4-3 deletion site	HXB2 deletion site
1408_09	4644-8700	4644-8710
2208_02	4851-7557	4851-7567
2278_24	3408-8591	3408-8601
2452_22	4861-5447	4861-5447
2531_09	4838-8479	4838-8489
2531_11	4317-8481	4317-8491
2531_19	3009-8629	3009-8639

Supplementary Table 6. Participant characteristics for CD4/CD8 coculture

Participant ID	Age	Sex	Time of therapy (years)	Viral load[a] (copies/mL)	Therapeutic regimen
PHPH	46	Male	3	47	3TC; DDI; EFV; TRU
MCCH	42	Male	3	20	NVP; KIV; ABC; FTC
SKTO	29	Male	3	47	NVP; TRU

3TC, lamivudine; ABC, abacavir; EFV, efavirenz; FTC, emtricitabine; NVP, nevirapine; DDI, didanosine; TRU, emtricitabine and tenofovir disoproxil fumarate; KIV, Kivexa; TRU, truvada

Supplementary Table 7. Sorting strategy used to obtain T_N and memory CD4+ T-cell subsets for each participant

Participants	T_N	T_{CM}	T_{TM}	T_{EM}
2026<br>2046<br>2115<br>2275<br>2518	CD45RO-/CD45RA+/CCR7+/CD27+/CD127+/CD95-	CD45RO+/CD45RA-/CCR7+/CD27+	CD45RO+/CD45RA-/CCR7-/CD27+	CD45RO+/CD45RA-/CCR7-/CD27-
2452<br>2302<br>2303<br>2241<br>2470<br>2469<br>2278<br>2286<br>1292	CD45RO-/CD27+/CCR7+/CD57-	CD45RO+/CCR7+/CD27+	CD45RO+/CCR7-/CD27+	CD45RO+/CCR7-/CD27-
1408<br>1756<br>2208<br>2274<br>2454<br>2531<br>2606<br>2647<br>2664<br>3632	HLA-DR-/CD45RA+/CCR7+/CD27+/CD57-/CD95-	HLA-DR-/CD45RA−/CCR7+/CD27+	HLA-DR-/CD45RA-/CCR7-/CD27+	HLA-DR-/CD45RA-/CCR7-/CD27-

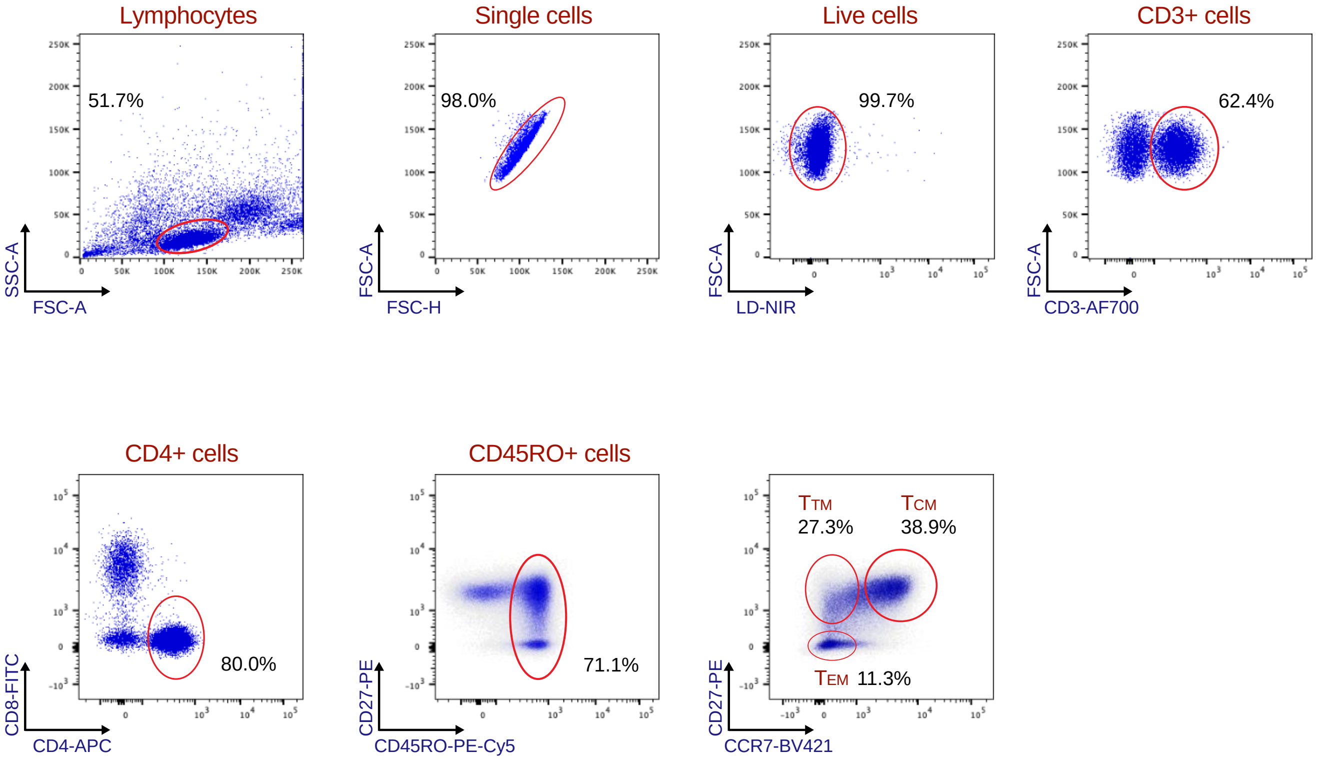

Supplementary Figure 1. Cell sorting strategy to isolate memory CD4+ T_{CM}, T_{TM} and T_{EM} cells from blood. (**A**) After excluding debris, doublets, and dead cells, live CD3+ CD4+ T-cells were gated. Memory cells were gated as CD45RO+, and CCR7 and CD27 then used to discriminate between the T_{CM} (CCR7+ CD27+), T_{TM} (CCR7- CD27+), and T_{EM} (CCR7- CD27-) subsets.

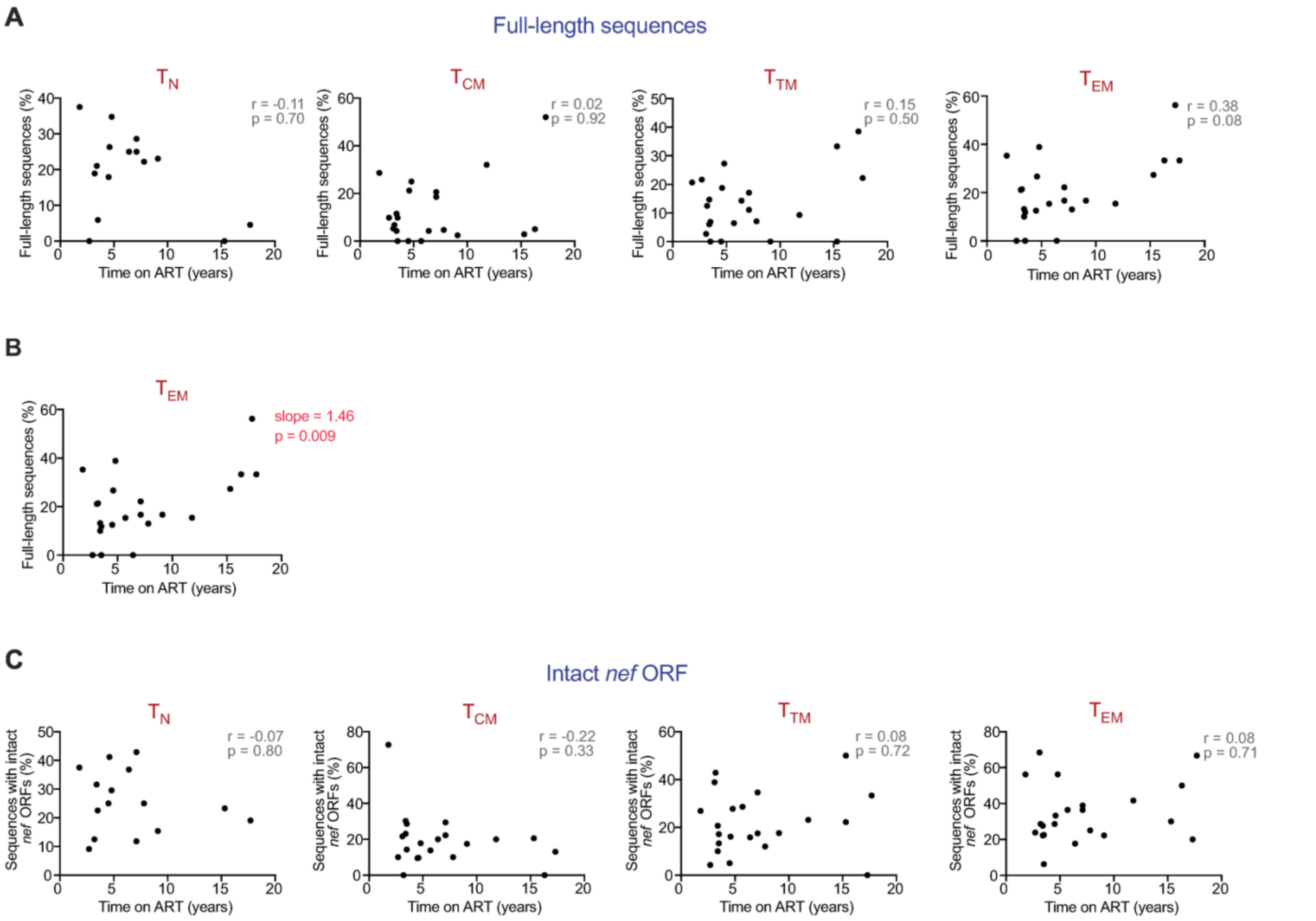

Supplementary Figure 2. The HIV-1 proviral landscape changes over time in T_N and memory CD4+ T-cell subsets.

Near-full-length HIV-1 proviral sequences were obtained by FLIPS from T_N and memory CD4+ T-cell subsets of participants on suppressive ART. (**A**) Spearman correlation analysis of the relationship between the percentage of full-length HIV-1 proviral sequences within each participant and time on ART (years) across T_N, T_{CM}, T_{TM}, and T_{EM} cells. **(B)** Linear regression analysis of relationship between the percentage of full-length HIV-1 proviral sequences within each participant and time on ART (years) in T_{EM} CD4+ T-cells. (**C**) Spearman correlation analysis of the relationship between the percentage of sequences with an intact *gag* ORF within each participant and time on ART (years) across T_N, T_{CM}, T_{TM}, and T_{EM} cells. Each data point represents the percentage of sequences obtained per participant. p≤0.05 values are shown in red. T_N, naïve; T_{CM}, central memory; T_{TM}, transitional memory; T_{EM}, effector memory; ORF, open reading frame.

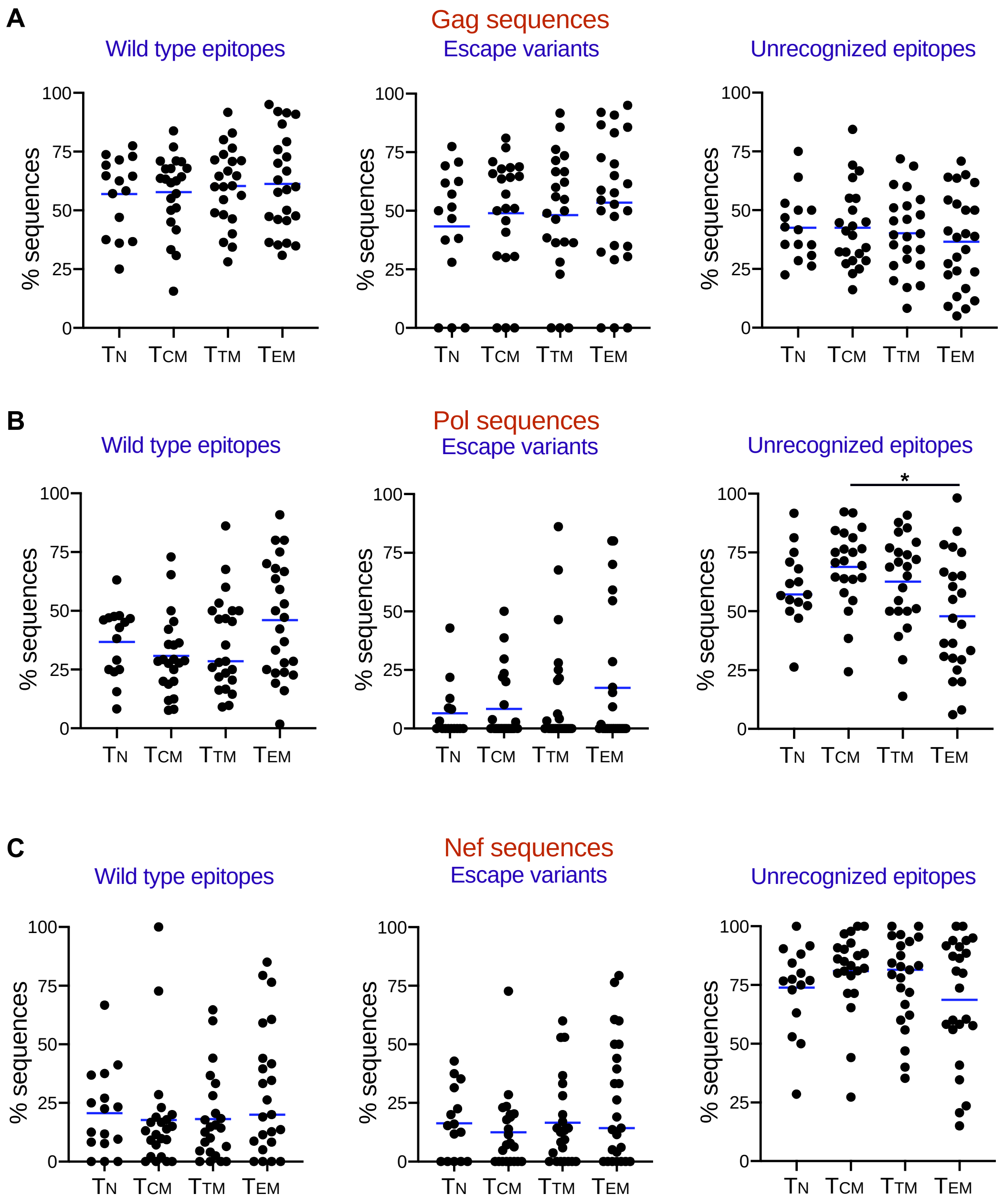

Supplementary Figure 3. Proportion of HIV-1 proviral sequences harboring CTL wild type epitopes, escape variants, and unrecognizable epitopes across T_N and memory CD4+ T-cells.

Near-full-length HIV-1 proviral sequences were obtained by FLIPS from T_N and memory CD4+ T-cell subsets of participants on suppressive ART. The percentage of HIV-1 proviral sequences harboring CTL wild type epitopes, escape variants, and unrecognizable epitopes were quantified for the viral proteins Gag (**A**), Pol (**B**), and Nef (**C**) across T_N, T_{CM}, T_{TM}, and T_{EM} cells. Each data point represents the percentage of sequences obtained per participant. Blue lines represent the mean value. Statistical significance was determined by Kruskal–Wallis followed by Dunn's post test. * $p \leq 0.05$. T_N, naïve; T_{CM}, central memory; T_{TM}, transitional memory; T_{EM}, effector memory.

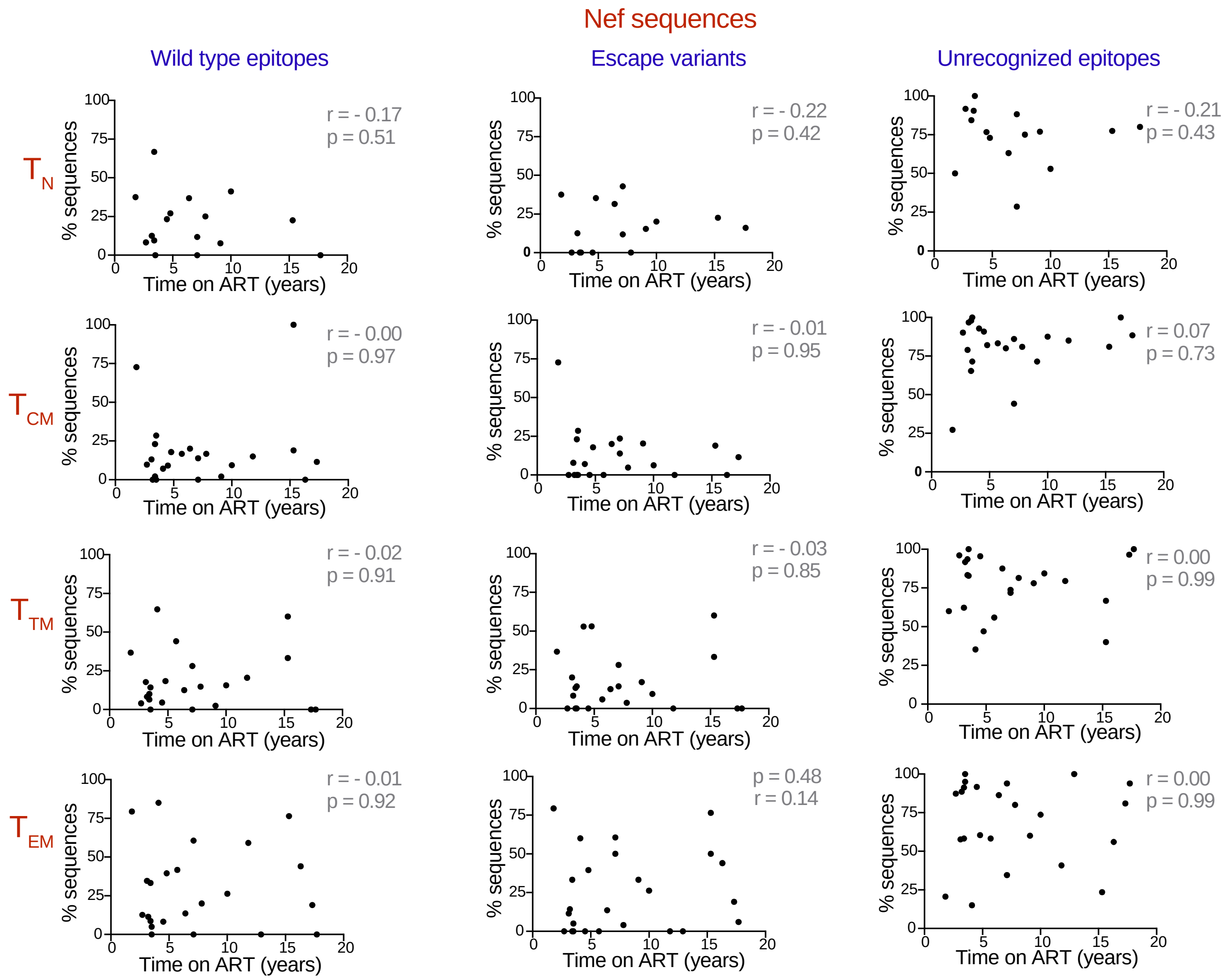

Supplementary Figure 4. The proportion of HIV-1 proviral sequences harboring CTL wild type, escape variants and unrecognizable epitopes for Nef does not correlate with time on ART in T_N and memory CD4+ T-cells.

Near-full-length HIV-1 proviral sequences were obtained by FLIPS from T_N and memory CD4+ T-cell subsets of participants on suppressive ART. (**A**) Spearman correlation analysis of the relationship between the percentage of HIV-1 proviral sequences harboring CTL wild type epitopes, escape variants, and unrecognizable epitopes for the viral protein Nef within each participant and time on ART (years) across T_N, T_{CM}, T_{TM}, and T_{EM} cells. Each data point represents the proportion of sequences obtained per participant. T_N, naïve; T_{CM}, central memory; T_{TM}, transitional memory; T_{EM}, effector memory.

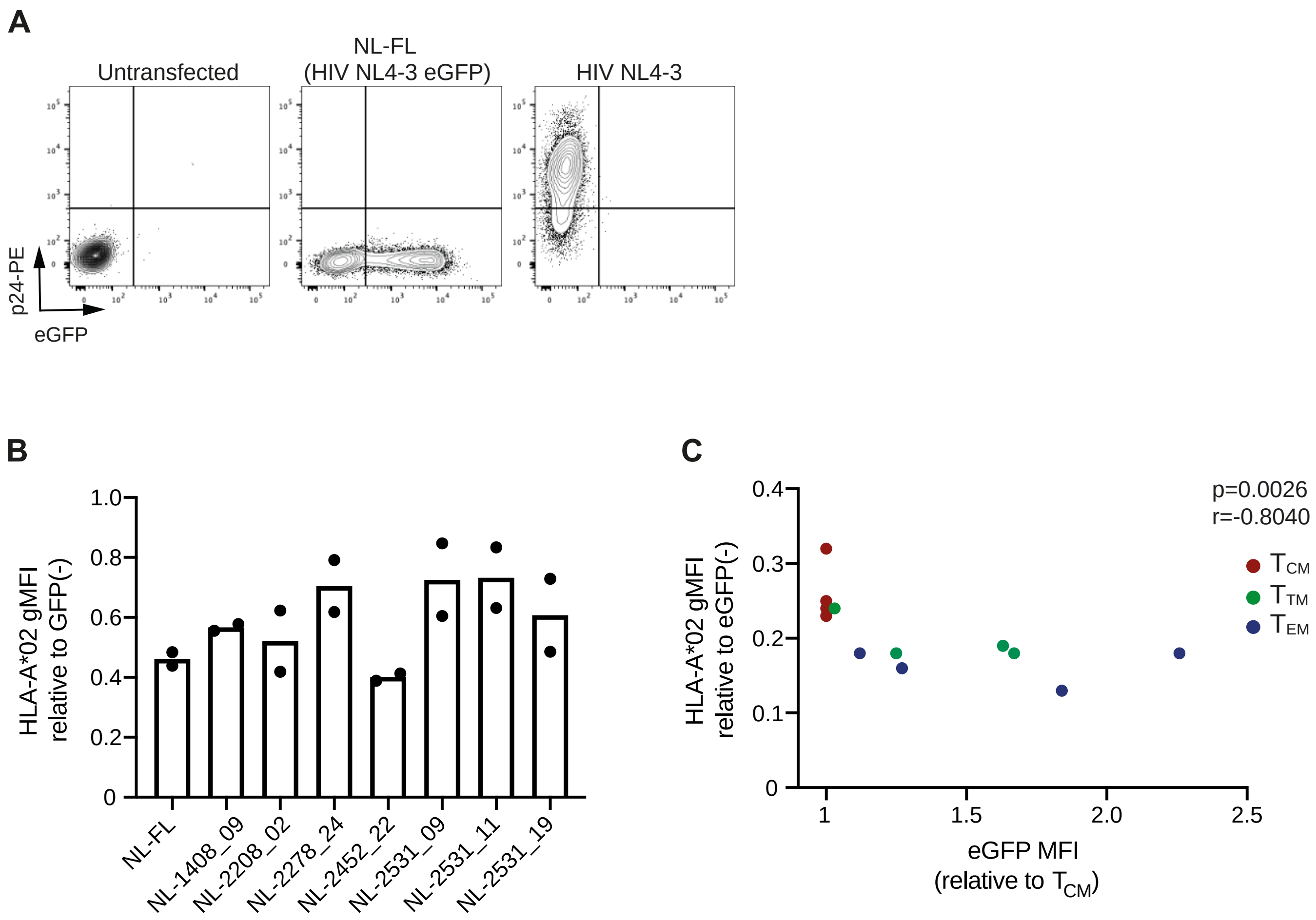

Supplementary Figure 5. Defective proviruses containing large internal deletions can express viral proteins.

(**A**) Flow cytometry of staining and compensation controls. Unstained HEK 293T cells transfected with an HIV-1 NL4-3 eGFP plasmid were used as a control for eGFP fluorescence (middle). HEK 293T cells transfected with HIV-1 NL4-3 plasmid (eGFP(negative) were stained with anti-p24-PE and used as a control for PE fluorescence (right). (**B**) Quantification of HLA-A*02 downmodulation in HEK 293T cells transfected with HIV-1 NL4-3-eGFP constructs containing internal deletions. Each data point represents values obtained from independent experiments. (**C**) Memory CD4+ T-cells were sorted from four HLA-A*02 positive HIV-1 negative donors and infected with HIV-1 NL4-3 eGFP. After day 5 post-infection, HLA-A*02 and eGFP expression were quantified by flow cytometry. Spearman correlation analysis between relative HLA-A*02 downmodulation and relative eGFP expression was performed. Each data point represents a single donor. T_N, naïve; T_{CM}, central memory; T_{TM}, transitional memory; T_{EM}, effector memory.

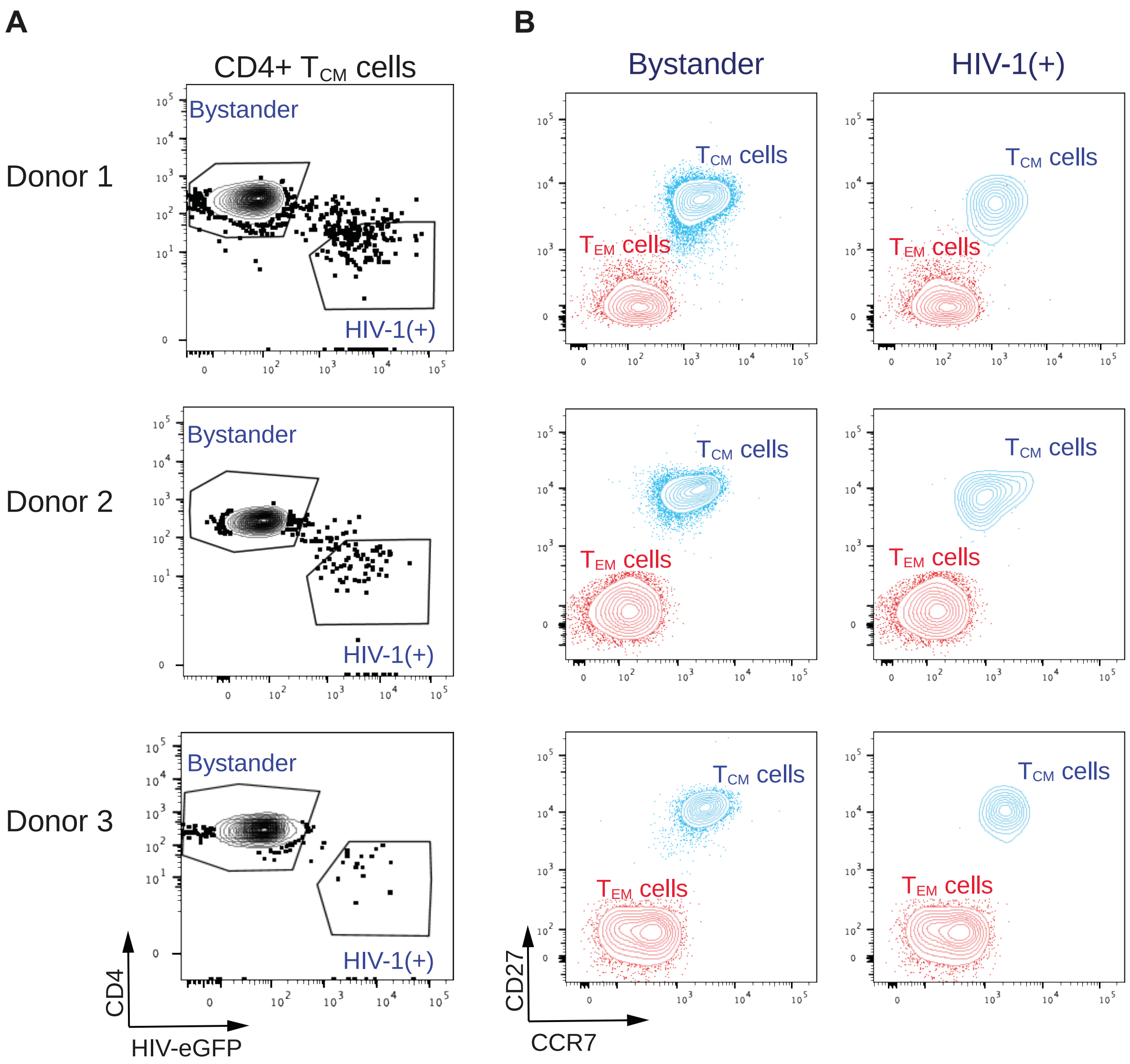

Supplementary Figure 6. T_{CM} cells express CD27 and CCR7 after HIV-1 infection.

T_{CM} CD4+ T-cells from 3 HIV-1 negative donors were sorted and infected with HIV-1 NL4-3 eGFP for 5 days. The expression of CD4 **(A)**, CD27 and CCR7 **(B)** was evaluated by flow cytometry. Bystander and HIV-1 positive cells were gated **(A)** and the expression of CD27 and CCR7 was quantified in both population **(B**; **blue).** T_{EM} cells from the same donor were used as control **(B; red).**
